# Pesticide Methoxychlor Promotes the Epigenetic Transgenerational Inheritance of Adult-Onset Disease through the Female Germline

**DOI:** 10.1371/journal.pone.0102091

**Published:** 2014-07-24

**Authors:** Mohan Manikkam, M. Muksitul Haque, Carlos Guerrero-Bosagna, Eric E. Nilsson, Michael K. Skinner

**Affiliations:** Center for Reproductive Biology, School of Biological Sciences, Washington State University, Pullman, Washington, United States of America; John A. Burns School of Medicine, United States of America

## Abstract

Environmental compounds including fungicides, plastics, pesticides, dioxin and hydrocarbons can promote the epigenetic transgenerational inheritance of adult-onset disease in future generation progeny following ancestral exposure during the critical period of fetal gonadal sex determination. This study examined the actions of the pesticide methoxychlor to promote the epigenetic transgenerational inheritance of adult-onset disease and associated differential DNA methylation regions (i.e. epimutations) in sperm. Gestating F0 generation female rats were transiently exposed to methoxychlor during fetal gonadal development (gestation days 8 to 14) and then adult-onset disease was evaluated in adult F1 and F3 (great-grand offspring) generation progeny for control (vehicle exposed) and methoxychlor lineage offspring. There were increases in the incidence of kidney disease, ovary disease, and obesity in the methoxychlor lineage animals. In females and males the incidence of disease increased in both the F1 and the F3 generations and the incidence of multiple disease increased in the F3 generation. There was increased disease incidence in F4 generation reverse outcross (female) offspring indicating disease transmission was primarily transmitted through the female germline. Analysis of the F3 generation sperm epigenome of the methoxychlor lineage males identified differentially DNA methylated regions (DMR) termed epimutations in a genome-wide gene promoters analysis. These epimutations were found to be methoxychlor exposure specific in comparison with other exposure specific sperm epimutation signatures. Observations indicate that the pesticide methoxychlor has the potential to promote the epigenetic transgenerational inheritance of disease and the sperm epimutations appear to provide exposure specific epigenetic biomarkers for transgenerational disease and ancestral environmental exposures.

## Introduction

Epigenetic transgenerational inheritance is defined as the germline transmission of epigenetic information and phenotypic change across generations in the absence of any direct environmental exposure or genetic manipulation [1], [2]. Exposure of a gestating female (F0 generation) also exposes the F1 generation fetus and germline within the fetus that will generate the F2 generation, such that the F3 generation progeny is the first transgenerational generation with no potential exposure [2], [3]. The critical window of exposure for the germline is during fetal gonadal sex determination when epigenetic reprogramming in the primordial germ cell undergoes a DNA demethylation and remethylation [1]. The environmental insults promote an apparent permanent alteration in the germline epigenome (DNA methylation) that escapes epigenetic reprogramming after fertilization, similar to an imprinted gene [4]. This germline epigenetic inheritance will alter the embryonic stem cell epigenome such that all cell types derived will have an altered epigenome and transcriptome and those somatic cell types sensitive to this altered epigenome and gene expression will be susceptible to develop adult onset disease across generations [5], [6]. A number of previous studies have shown environmental toxicants including the fungicide vinclozolin [2], [4], plastics (bisphenol A and phthalates) [7], pesticide (DEET and permethrin) [8], dioxin [9], hydrocarbons (jet fuel) [10], and dichlorordiphenyltrichloroethane (DDT) [11] promote the epigenetic transgenerational inheritance of adult onset disease and sperm epimutations [12]. Interestingly, the transgenerational epigenetic alterations (epimutations) in sperm appear exposure specific and may be useful as biomarkers of ancestral toxicant exposure and susceptibility to develop transgenerational adult onset disease [12].

Methoxychlor is considered a model environmental endocrine disruptor with estrogenic and anti-androgenic activity [13]. It has been used as an approved insecticide and pesticide to replace DDT for application on agricultural crops and livestock since its commercial production in the USA in 1946 [14]. Contamination of food with methoxychlor has been previously observed [15]. The toxic effects of methoxychlor in animal studies have been reviewed and they include adverse effects on fertility, early pregnancy and in utero development in females, as well as altered social behavior in males after prenatal exposure [13]. A two-generation rat study examined methoxychlor's estrogenic and reproductive toxicity and found suppression of body weights, prolonged estrous cycles, reduced fertility, decreased numbers of implantation sites and newborns, decreased ovary weights, increased incidence of cystic ovary, increased uterine weights, delayed preputial separation, reduced sperm counts, and altered reproductive organ weights [16]. Methoxychlor inhibits testosterone formation in rats [17], [18], [19], [20], causes disruption of adult male reproductive function following transient exposure during sexual differentiation in rats [21], [22], and alteration of mammary gland development in male rats [23], [24]. Induction of testis abnormalities in the F1 and F2 generations following exposure has been recorded with prenatal exposure to methoxychlor [25]. Methoxychlor is carcinogenic for the liver, testis, ovary, spleen, blood vessels, pituitary, adrenals and mammary glands in rodents [26]. Neonatal direct exposure to methoxychlor can influence pregnancy [27], [28], ovarian and hypothalamic function [29], [30], reproductive behavior [31], prostate development [32], thymus development [33], and testis development [34]. Developmental methoxychlor exposure results in reduced ovulation and fertility and premature aging in rats [35] and altered reproductive and startle behaviors [36], [37]. Neuroendocrine effects of prenatal exposure to methoxychlor have been reported [38]. A reduction in estrogen receptor beta gene expression in sheep hypothalamus was found after prenatal exposure to methoxychlor [39]. Lifelong effects on neuroendocrine gene expression and premature reproductive aging occur following early life exposure to methoxychlor [38]. In addition, epigenetic alterations in adult ovarian or hypothalamic genes are induced by fetal and neonatal direct exposure to methoxychlor [38], [40]. Exposure of gestating F0 generation females to methoxychlor during fetal gonadal sex differentiation in the rat caused transgenerational male testis effects including increased spermatogenic cell apoptosis, decreased sperm counts, and decreased sperm motility [2]. Methoxychlor treatment of pregnant mice decreased the mean sperm concentrations by 30% and altered the methylation pattern of all the imprinted genes tested in the F1 offspring [41]. Recently, methoxychlor has been shown to promote female reproductive disease and epigenetic changes in ovarian tissues and function [40], [42], [43]. The toxicity profile of methoxychlor in humans revealed death, systemic (aplastic anemia), cardiovascular (low blood pressure), and neurological (blurred vision, dizziness and paresthesia) effects, and cancer (leukemia) [44].

The current study investigates methoxychlor's potential to promote the epigenetic transgenerational inheritance of adult-onset disease in both males and females of subsequent generations following the transient exposure of an F0 generation gestating female during fetal gonadal sex determination. The parental origin of the germline transmission and germline epimutations are also investigated. This study used a dose of 200 mg/kg body weight for methoxychlor (4% of rat oral LD50) for administration to gestating rats. This is within the range of high environmental exposure dose for methoxychlor [45]. No direct exposure toxic effects were anticipated in the current study or observed. The current study was not performed as a risk assessment study, but to investigate the potential and mechanisms involved in the transgenerational actions of methoxychlor. Previous studies demonstrated that the transgenerational actions of the pesticide DDT promoted obesity and other disease, so a comparison with the actions of methoxychlor was made [11]. In the current study, diseases of the testis, prostate, kidney, ovary and uterus, as well as tumor development, abnormal puberty onset and obesity were evaluated in 10–12 month old F1 and F3 generation control and methoxychlor lineage rats. This study further documented sperm epimutations that are associated with adult onset disease and found a methoxychlor specific pattern of DNA methylation change in the sperm. These sperm epimutations in the F3 generation methoxychlor lineage are unique in comparison to a number of other exposure specific epimutation signatures including DDT. These epigenetic alterations may be useful as biomarkers of ancestral methoxychlor exposure and transgenerational adult-onset disease.

## Results

### Transgenerational adult-onset disease analysis

The epigenetic transgenerational actions of methoxychlor administered to female rats during day 8 to 14 of gestation were investigated. This F0 generation transient exposure was the only exposure. F1 generation offspring were bred to generate the F2 generation which was bred to generate the F3 generation for both the vehicle dimethylsulfoxide (DMSO) control and methoxychlor lineages [2], [3], [4], [7], [8], [9], [10], [11], [12]. No sibling or cousin breedings were used to avoid any inbreeding artifacts. The F1 and F3 generation rats of control and methoxychlor lineages were euthanized at 10–12 months of age. Body weights were measured and testis, prostate, kidney, ovary and uterus histopathology were examined. To assess if there were any toxic effects from embryonic exposure to methoxychlor the F1 generation body weights and organ weights were measured (Tables S1A and S1B). The body weights of the F1 and F3 generation methoxychlor lineage females were unaltered compared to controls. Following methoxychlor exposure the kidney weights in females and males of the F1 generation were increased. The ovarian and uterine weights were unaffected in the F1 generation. The testis weight decreased in the F1 generation methoxychlor lineage. The prostate, seminal vesicle and epididymal weights were unaltered in F1 generation. In addition, serum sex steroid hormone concentrations were measured in the F3 generation to assess any endocrine alteration. Serum estradiol concentrations in F3 generation females during diestrus phase or proestrus-estrus phase were unaffected (Figure S1). Serum testosterone concentrations in F3 generation males were unchanged (Figure S1, panel C). Observations indicate that overall there were no major F1 generation toxicity effects and no F3 generation endocrine effects from methoxychlor exposure.

**Figure 1:**
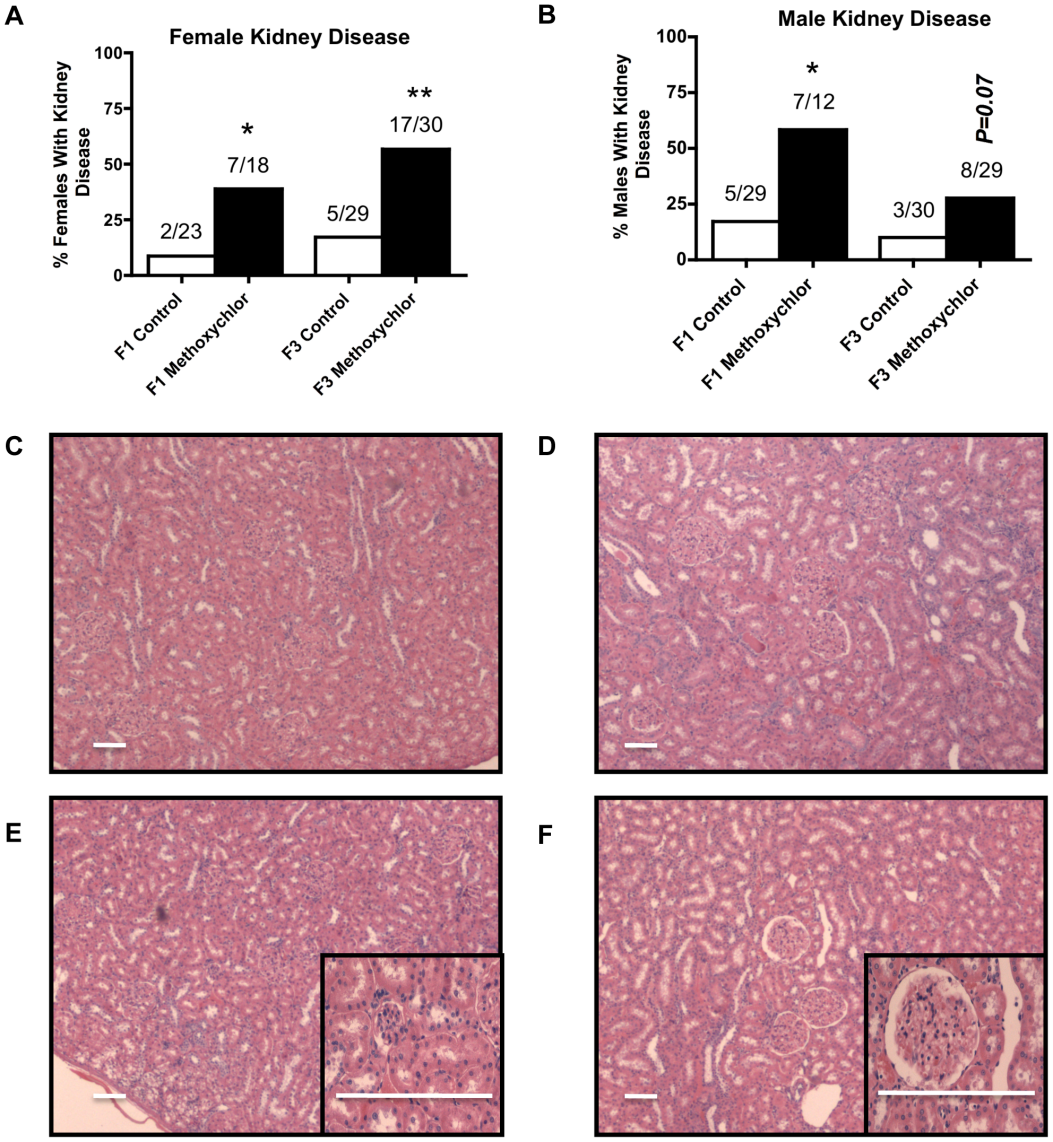
Ancestral exposure to methoxychlor and transgenerational kidney disease. Percentages of females (panel A, C, E) and males (panel B, D, F) with kidney diseases in the F1 and F3 generation control (open bars) and methoxychlor (black bars) lineages. The number of diseased rats / total number of rats in each lineage are also shown (* P<0.05; ** P<0.01).

The incidences of kidney disease in methoxychlor lineages are presented in Figure 1, panels A (females) and B (males) respectively. Kidney disease was characterized by either the presence of an increased number of proteinaceous fluid filled cysts or reduction in size of glomeruli or abnormality of Bowman's capsule thickness (Figure 1, panels C and D [control]; panels E and F [methoxychlor]). Previously these morphological abnormalities have been shown to be associated with an abnormal blood-urea-nitrogen (BUN) in the animals [46]. There was an increase in kidney disease in F1 and F3 generation females of methoxychlor lineages (Figure 1, panel A). The F1 generation males of methoxychlor lineages showed an increase in kidney disease, while the F3 generation methoxychlor lineage males manifested an incidence of kidney disease that approached a significant increase (Figure 1, panel B).

The incidences of testis and prostate diseases in methoxychlor lineages are presented in Figure S2, panels A and B respectively. Testis disease was characterized by the presence of histopathology including azoospermic and atretic seminiferous tubules, presence of vacuoles in basal regions of seminiferous tubules, sloughed cells in the lumen of seminiferous tubules and lack of seminiferous tubule lumen (Figure S2, panel C [control]; panel E [methoxychlor]). There were no significant increases in testis disease in the F1 or F3 generation one year old males of methoxychlor lineages. To further study testis disease the number of apoptotic spermatogenic cells was examined by TUNEL analysis. The number of apoptotic spermatogenic cells did not significantly increase in the F1 or the F3 generation rats of methoxychlor lineages (Figure S3). Therefore spermatogenic defects that were present in vinclozolin lineage F3 generation rats [2] were not observed in F3 generation rats of methoxychlor lineages. Prostate disease was characterized by atrophic prostatic duct epithelium (Figure S2, panel D [control]; panel F [methoxychlor]. The incidence of prostate disease also did not significantly increase in the F1 or the F3 generation males of methoxychlor lineage.

**Figure 2:**
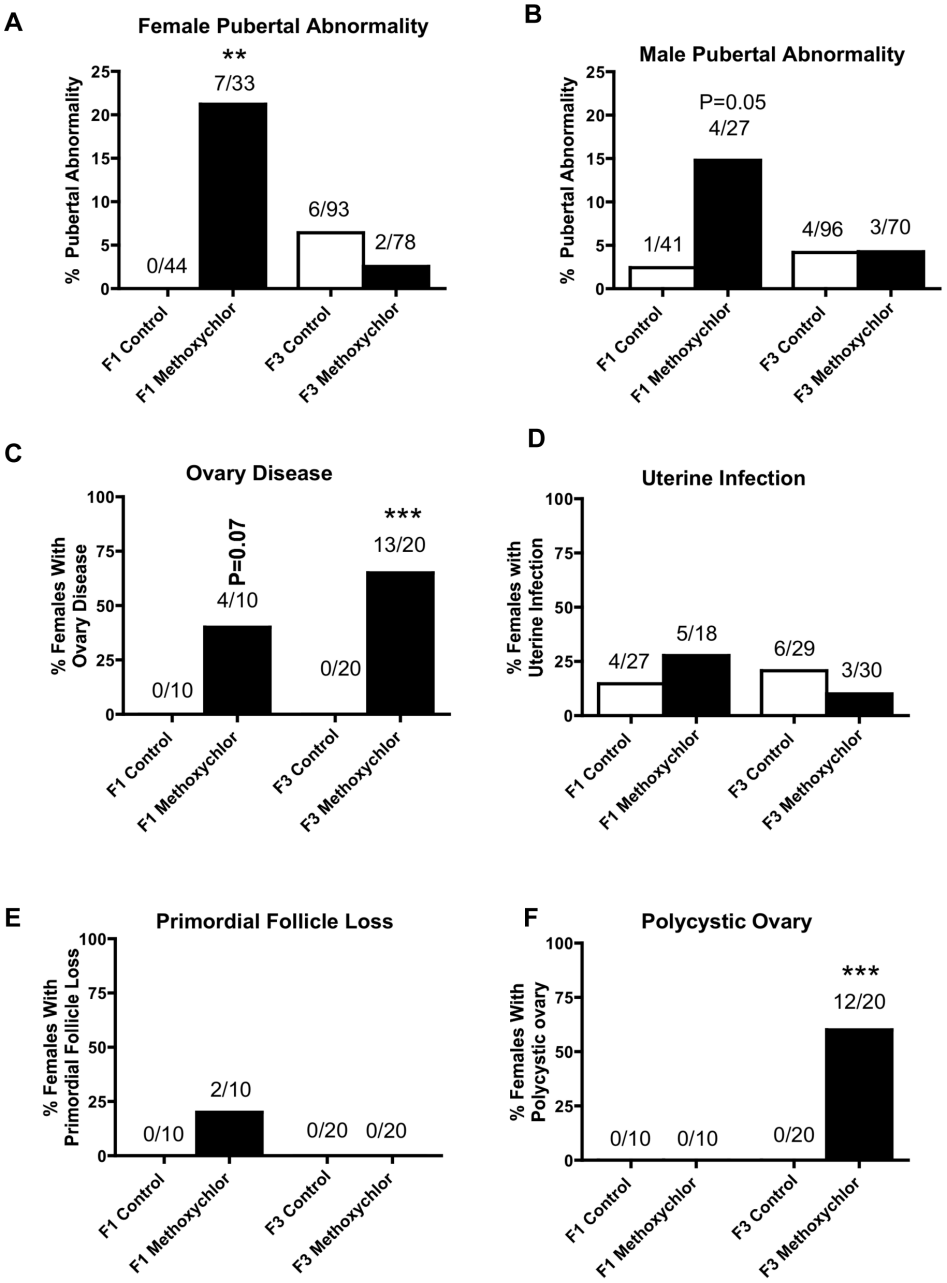
Ancestral exposure to methoxychlor and transgenerational pubertal abnormalities, and ovarian and uterine diseases. Percentages of females F1 and F3 generation (panel A) and males (panel B) with pubertal abnormality or females with ovarian disease (panel C) or uterine infection (panel D). Percentages of the F1 and F3 generation females with primordial follicle loss (panel E) and polycystic ovary disease (panel F) in control and methoxychlor lineages. The number of diseased rats / total number of rats in each lineage are also shown. (** P<0.01; *** P<0.001).

The incidences of pubertal abnormalities in females (Figure 2, panel A) and males (Figure 2, panel B), ovary disease (Figure 2, panel C) and uterine infection (Figure 2, panel D) are presented. There was no increase in pubertal abnormalities in either the F1 or the F3 generation females or males of methoxychlor lineages. This included an assessment of both premature or delayed pubertal conditions as previously described [46], [47]. In contrast, the ovarian disease incidence increased both in the F1 and F3 generations of methoxychlor lineage (Figure 2, panel C). The ovarian disease was presented as a primordial follicle pool decrease or polycystic ovary disease [48]. The primordial follicle ovarian pool was shown to have a reduction in the number of primordial follicles per ovary section. The polycystic ovarian disease was determined by an increase in the number of small and large cysts (Figure 2 panel F). In the F1 generation the ovarian disease in methoxychlor lineage was characterized by a primordial follicle pool decrease and a zero incidence of ovarian cysts. However, the F3 generation methoxychlor lineage ovarian disease was primarily characterized by the presence of ovarian cysts. The incidence of uterine infection did not significantly increase in the F1 or F3 generation rats of methoxychlor lineage (Figure 2, panel D). Uterine infection was determined by the enlargement of uterus, accumulation of foul-smelling dark discolored purulent material and presence of inflammation within the uterine horns [11]. In addition, no significant change in uterine morphology was observed, as previously described [47].

**Figure 3:**
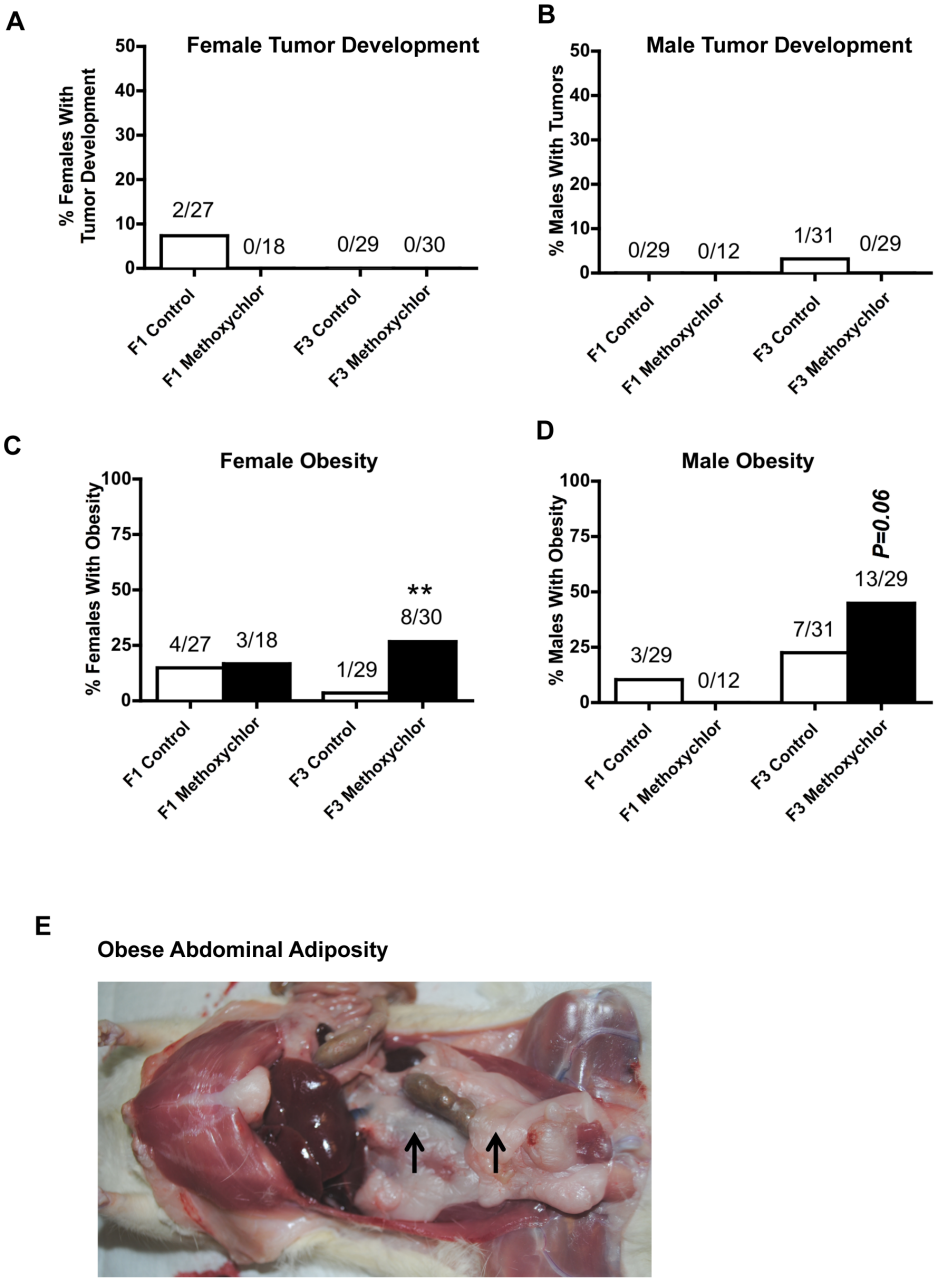
Ancestral exposure to methoxychlor and transgenerational tumor development and transgenerational obesity. Percentages of F1 and F3 generation females (panel A) or males (panel B) with tumor development and percentages of females (panel C) or males (panel D) with obesity. Representative abdominal adiposity for obese (panel E) animals is presented with arrows indicating the excessive dorsal abdominal and retro-peritoneal adiposity distribution. The number of diseased rats / total number of rats is shown above the respective bar graphs (** P<0.01).

The incidence of tumor development in females and males, and the incidence of obesity in females and males are shown in Figure 3. The primary tumors previously observed in transgenerational models were mammary gland tumors [46]. There was no significant increase in tumor development in either females or males of the F1 and the F3 generation in methoxychlor lineage (Figure 3, panels A and B). The incidence of obesity in females and males did not increase in the F1 generation methoxychlor lineages (Figure 3, panels C and D). The incidence of obesity increased in females of the F3 generation of methoxychlor lineage (Figure 3, panel C). The incidence of obesity tended to increase in the F3 generation males of methoxychlor lineage (p<0.06; Figure 3 panel D). The occurrence of obesity was determined by the excessive accumulation of subcutaneous and intra-abdominal adipose tissue and weight gain [11] (Figure 3, panel E). Although the average weight of all animals combined for a generation lineage was not altered, (Table S1), comparison of obese versus non-obese animal weights showed alterations. The mean weight of the methoxychlor lineage obese males (553±10 g) and females (317±9 g) was compared to mean weight of non-obese males (497±12 g) and females (284±8 g) respectively and found to be statistically different (p<0.05).

**Figure 4:**
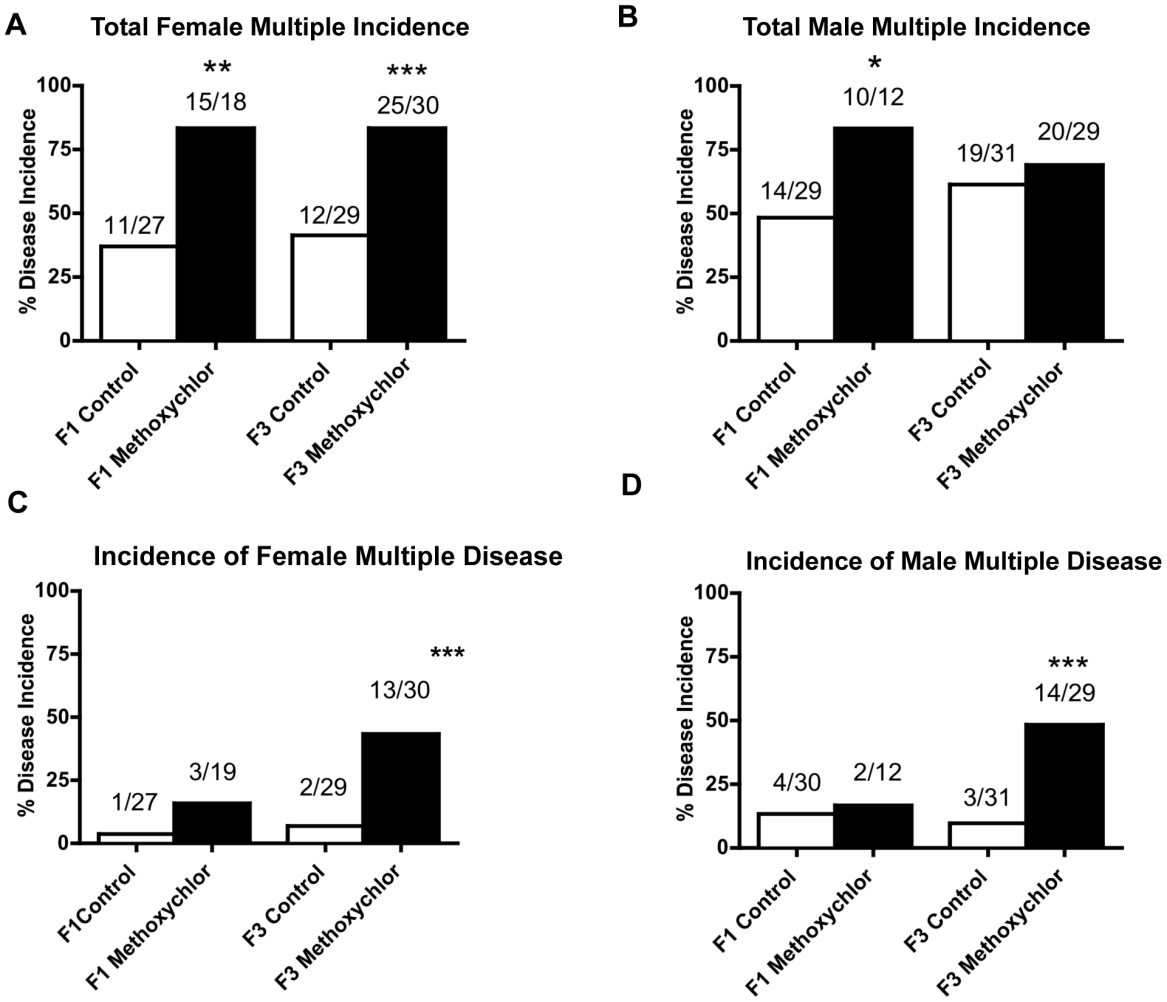
Ancestral exposure to methoxychlor and adult-onset transgenerational disease in rats. Incidences of F1 and F3 generation total female disease (panel A), total male disease (panel B), female multiple disease (panel C) and male multiple disease (panel D) in control and methoxychlor lineages. The number of diseased rats / total number of rats is shown above the respective bar graphs (* P<0.05; ** P<0.01; *** P<0.001).

The incidence of diseases in individual rats from control and methoxychlor lineage is presented in Table S2A (F1 generation females), Table S2B (F1 generation males), Table S3A (F3 generation females) and Table S3B (F1 generation males). The incidence of total diseases per rat increased in both the F1 and the F3 generation females of methoxychlor lineage (Figure 4, panel A). The incidence of total disease per rat increased in the F1 generation males, but it did not increase in the F3 generation males of methoxychlor lineage (Figure 4, panel B). The incidence of multiple disease did not increase in the F1 generation females or males (Figure 4 panels C and D). The incidence of multiple diseases per rat increased in the F3 generation females of methoxychlor lineage (Figure 4, panel C). The incidence of multiple diseases also increased in the F3 generation males of methoxychlor lineage (Figure 4, panel D). Exposure to methoxychlor in F0 generation females therefore increased the overall incidence of adult onset diseases both in the F1 and F3 generation rats. Therefore, female gestating ancestors exposed to methoxychlor transmitted kidney disease, ovarian disease and obesity to their unexposed F3 generation descendants. Methoxychlor was found to induce the epigenetic transgenerational inheritance of adult-onset disease.

**Figure 5:**
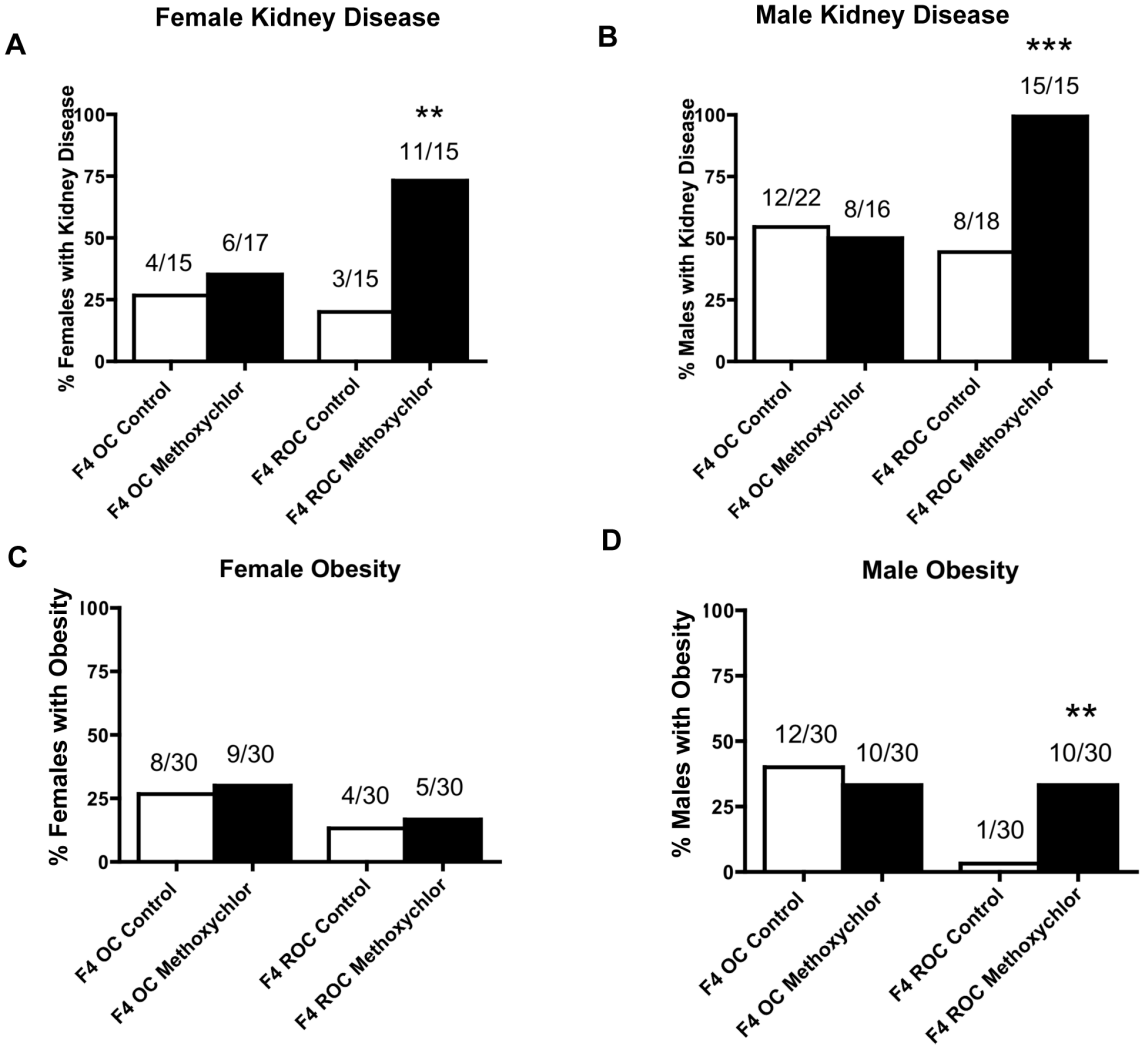
Ancestral exposure to methoxychlor promoted adult-onset transgenerational diseases in F4 generation reverse outcross offspring showing female germline transmission. Incidences of kidney disease in females (panel A), in males (panel B), obesity in females (panel C) and in males (panel D) of the F4 generation outcross (OC) or reverse outcross (ROC) offspring of the control and methoxychlor lineages.

The parental origin of the germline transmission was investigated to determine if the epigenetic transgenerational inheritance of adult onset diseases is transmitted through the male or female germline [2], [11]. An outcross of a control or methoxychlor lineage F3 generation male with a wildtype female was examined to assess sperm transmission. Reverse outcrosses from the F3 generation control or methoxychlor lineage females with wildtype males were examined to assess oocyte transmission. The F4 generation outcross and reverse outcross offspring were maintained until the age of 10 months and disease analysis was performed and compared with the F3 generation animals (Tables S4, S5 and S6). The incidence of kidney disease in females (Figure 5, panel A), in males (Figure 5, panel B), obesity in females (Figure 5, panel C) and in males (Figure 5, panel D) in the F4 generation control and methoxychlor lineages are presented. The first set of graphs in each panel is from the outcross (OC) (F3 males bred with wild type females) and the second set of graphs in each panel is from the reverse outcross (ROC) (F3 females bred with wild type males). The incidence of kidney disease in the outcross F4 generation females of methoxychlor lineage was unaltered, while that of the reverse outcross females increased (Figure 5, panel A). Similarly, the incidence of kidney disease in the outcross F4 generation males of methoxychlor lineage was unchanged (Figure 5, panel B), but that of the reverse outcross males increased (Figure 5, panel B). The incidence of obesity in the F4 generation outcross and reverse outcross females of methoxychlor lineage were unaltered (Figure 5, panel C). The incidence of obesity in the F4 generation outcross males was unchanged while that of the reverse outcross males was increased (Figure 5, panel D). Therefore, the female obesity was not transmitted by either germline alone, but the male obesity was transmitted only through the female germline.

**Figure 6:**
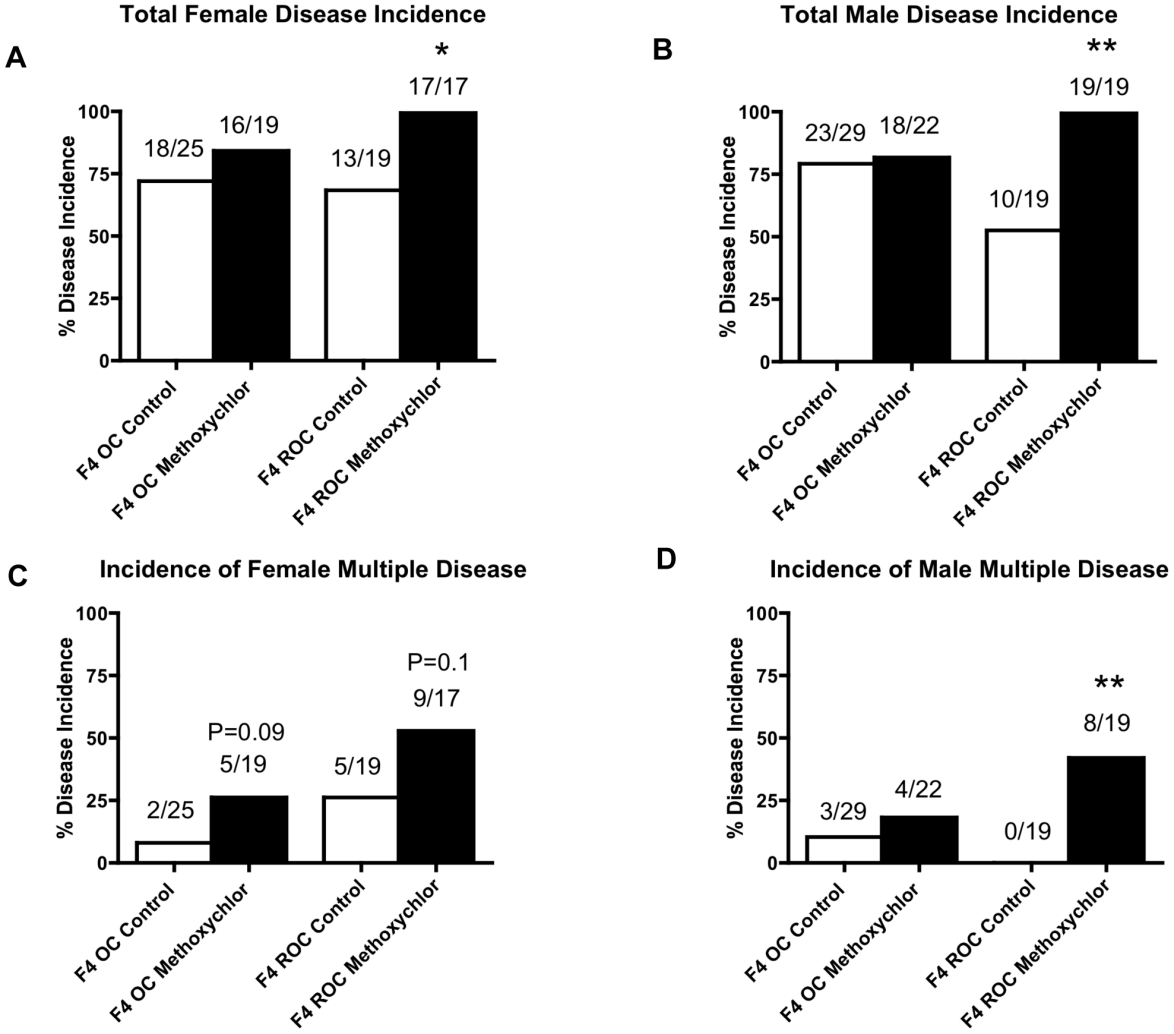
Ancestral exposure to methoxychlor promoted adult-onset transgenerational diseases in F4 generation reverse outcross offspring increasing total female disease, total male disease and multiple male disease incidences. Incidences of total disease in females (panel A), in males (panel B), multiple female disease (panel C), and multiple male disease (panel D) in the F4 generation outcross (OC) or reverse outcross (ROC) offspring of the control and methoxychlor lineages.

The incidence of total and multiple disease in the F4 generation outcross and reverse outcross offspring of the control and the methoxychlor lineages are shown in Figure 6 and Tables S5 and S6. The incidence of the total disease in the F4 generation outcross females of the methoxychlor lineage were unaltered (Figure 6, panel A), but that of the reverse outcross females increased (Figure 6, panel A). The incidence of the total disease in the F4 generation outcross males of the methoxychlor was also unaltered but the incidence in the reverse outcross males were increased (Figure 6, panel B). The incidence of the multiple disease in the F4 generation outcross and reverse outcross females of the methoxychlor lineage tended to increase (Figure 6, panel C). The incidence of multiple disease in the F4 generation reverse outcross males of the methoxychlor were increased but that of outcross males did not increase (Figure 6, panel D). The incidence of testis disease, ovary disease, uterine disease, female and male pubertal abnormalities (i.e. premature or delayed) and tumors in both females and males were evaluated and there was no increase in any of these disease incidence in the F4 generation outcross or reverse outcross methoxychlor lineages (data not shown). A combination of both male and female exposure lineage germlines appear to be needed for these pathologies to manifest. Interestingly, observations indicate that the transmission of the increased incidence of kidney disease in both females and males, obesity in males, total disease in both females and males, and multiple disease in males occurs through the female germline.

### Epigenetic Transgenerational Transmission of Sperm Epimutations

**Figure 7:**
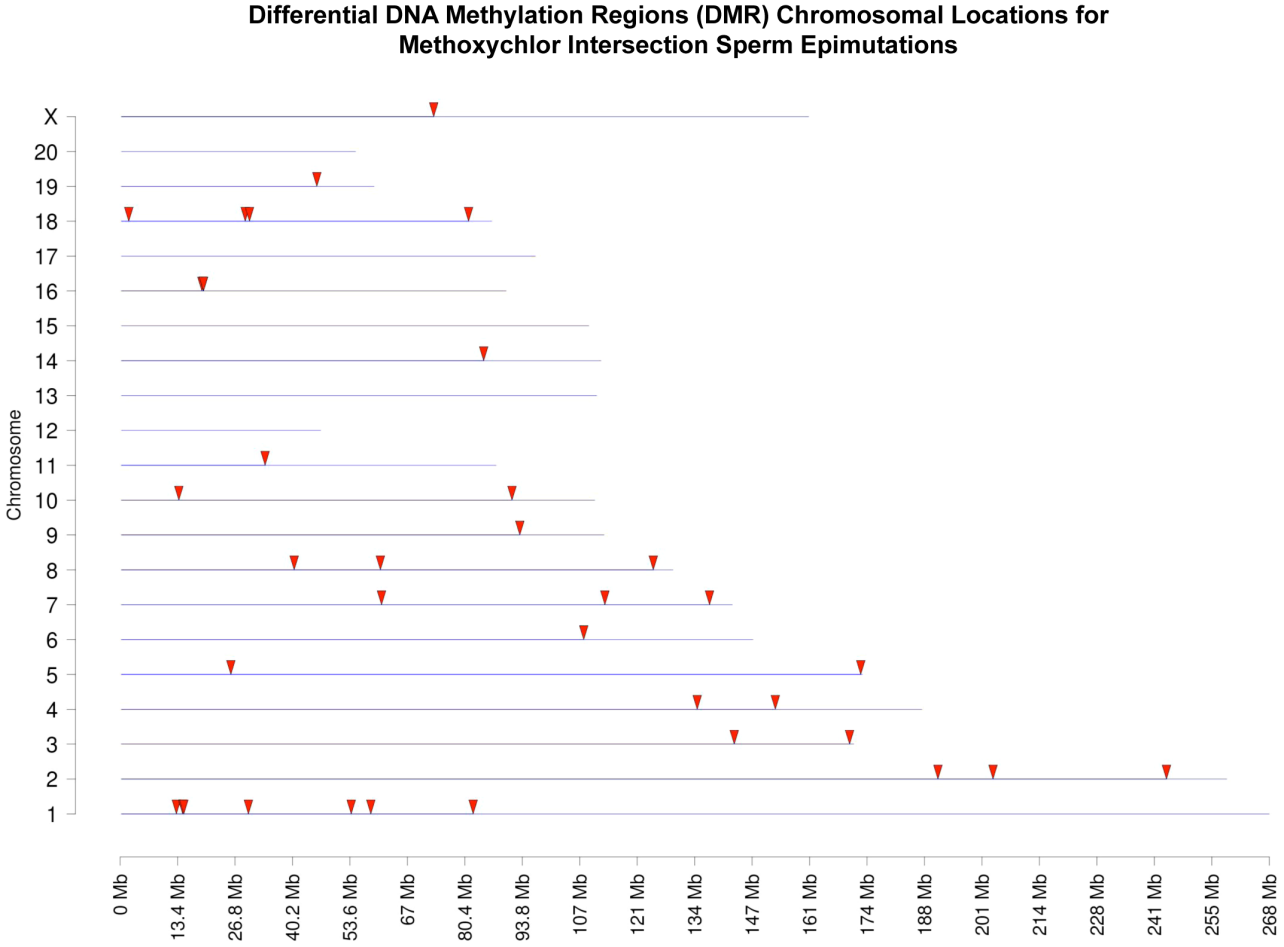
Ancestral exposure to methoxychlor and transgenerational epigenetic changes and induced sperm intersection epimutations. Chromosomal locations for regions with transgenerational change in DNA methylation (arrowheads). There were 37 differentially methylated regions in sperm DNA from methoxychlor lineage rats compared to control lineage rats for all three experiments.

**Figure 8:**
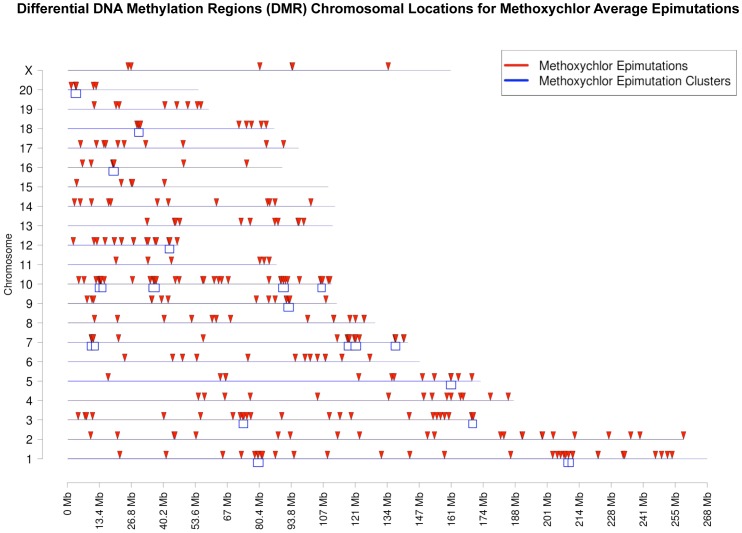
Ancestral exposure to methoxychlor and transgenerational epigenetic changes and induced sperm average epimutations. Chromosomal locations for regions with transgenerational change in DNA methylation (arrowheads). There were 311 differentially methylated regions in sperm DNA from methoxychlor lineage rats compared to control lineage rats for all experiments. The box under the line at each chromosome represents a statistically significant over-representation (cluster) of epimutations.

The methoxychlor induced epigenetic transgenerational inheritance of disease requires the germline transmission of epimutations. Previous studies have demonstrated F3 generation sperm develop differential DNA methylation regions (DMR) induced by a variety of different environmental toxicants [4], [7], [8], [9], [10], [11], and these epimutation signatures are unique to the specific environmental exposure [12]. The current study investigated the epimutations induced by methoxychlor in the F3 generation sperm. Three different experiments and comparisons with each involving different litters were used. The F3 generation control (DMSO) and methoxychlor lineage sperm were collected and DNA isolated for use in a methylated DNA immunoprecipitation (MeDIP) with an antibody to methyl-cytosine. The samples were pooled and then analyzed on a genome-wide rat promoter tiling array chip (MeDIP-Chip) as previously described [4], [7], [8], [9], [10], [11], [12]. Those DMR in the methoxychlor lineage sperm that were statistically significant (p<10^−5^) from the control lineage sperm in a competitive hybridization, and were similar between all three different experiments, were identified and termed “intersection” epimutations with a total of 37 presented in Figure 7 and Table 1. These stringently selected intersection epimutations were the most reproducible between experiments and statistically significant. Using an average of three different experiments a total of 311 “average” epimutations were identified and their chromosomal locations are indicated in Figure 8. The list of all methoxychlor induced sperm epimutations are presented in Table S7. A number of the average epimutations appeared to cluster into similar chromosomal regions so a cluster analysis was performed as previously described [6] and a number of chromosomal regions were identified to have a statistically significant over-representation of epimutations in approximately two megabase regions with approximately five epimutations (Figure 8 and Table S8). These average epimutation clusters may represent regions of the chromosomes more sensitive to epigenetic regulation.

**Table 1:** Methoxychlor induced intersection sperm epimutations.

Chromosome	cSTART	cSTOP	TSS	NCBI_geneID	GeneName
chr1	13103114	13104047	13106940	292944	Reps1
chr1	14568479	14569659	14568461	293012	Olig3
chr1	14842235	14843892	14846368	116465	Ifngr1
chr1	29897247	29897847	29897535	252881	Exoc3
chr1	53872637	53873237	53872493	100125364	LOC100125364
chr1	58442194	58442794	58442219	117281	Ppp2r1a
chr1	82306401	82307001	82306422	308451	Itpkc
chr2	190656177	190656777	190653151	689432	Mrps21
chr2	203497542	203498142	203496812	81869	Gstm7
chr2	243932137	243932737	243932220	64157	Ddah1
chr3	143178668	143179268	143178674	24888	Bcl2l1
chr3	170065543	170066143	170064582	296469	Nkain4
chr4	134540928	134541528	134541533	65275	Gpr27
chr4	152753789	152754389	152757539	405214	Olr825
chr5	25809500	25810100	25809542	297902	Gem
chr5	172666254	172667034	172666511	298686	Ccnl2
chr6	108089573	108090173	108089348	314304	Acot3
chr7	60939714	60940314	60937868	314879	Xpot
chr7	113019994	113020594	113018824	353498	Cyp11b3
chr7	137413059	137413659	137412091	300208	Ddx23
chr8	40573699	40574299	40573738	498140	RGD1560888
chr8	60673757	60674357	60673400	315696	Snx33
chr8	124305365	124305965	124303798	301059	Myd88
chr9	93184961	93185561	93185687	301626	Pdcd1
chr10	13680615	13681300	13682999	29740	Dci
chr10	91363947	91364622	91364902	303567	Tmub2
chr11	33787230	33787830	33785529	245975	Setd4
chr14	84759189	84759789	84761365	305478	Rnf215
chr16	19042902	19043502	19042062	290641	Rpl18a
chr16	19413846	19414446	19413528	498606	LOC498606
chr16	19426339	19426939	19425400	64156	Uba52
chr18	2000825	2001425	2001139	291794	Snrpd1
chr18	29156910	29157510	29153944	25433	Hbegf
chr18	30151115	30151715	30150686	291653	Pcdhb6
chr18	81234782	81235382	81232949	291394	Cndp2
chr19	45861256	45861856	45861454	54267	Maf
chrX	73108036	73108636	73108016	100126191	Rab1b

**Figure 9:**
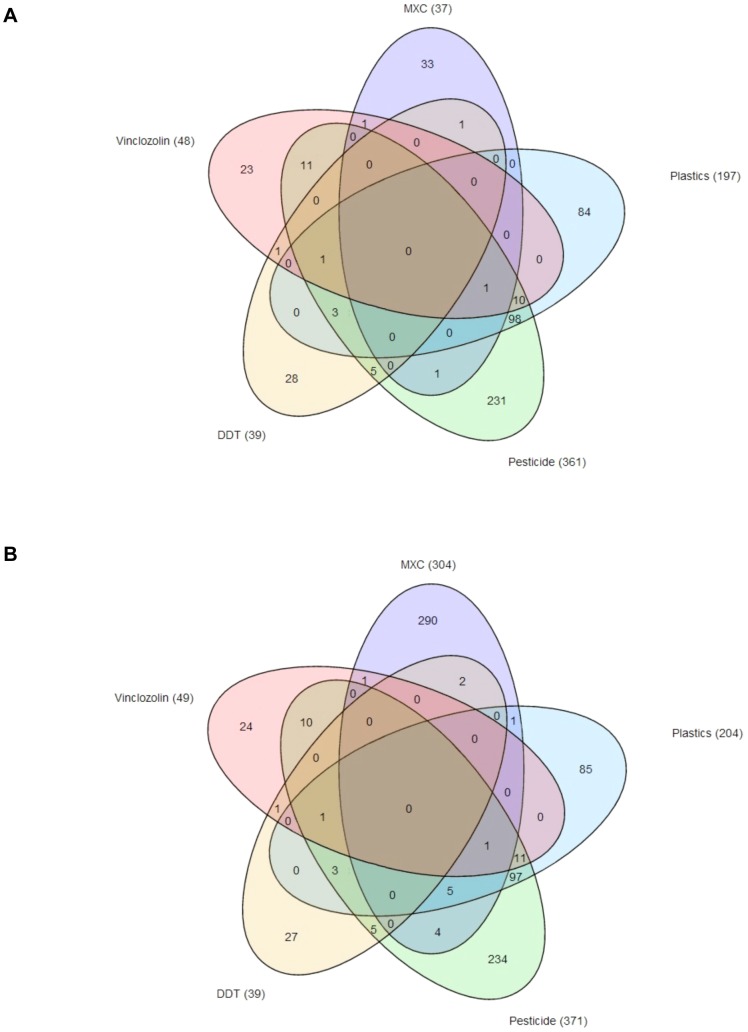
Exposure specific transgenerational sperm epimutations in methoxychlor, vinclozolin, DDT, pesticide (DEET and permethrin) and plastics (BPA and phthalates). A Venn diagram indicating the total number of intersection epimutations (A) and average epimutations (B) involving overlap between the various epimutation exposure data sets. Total number of epimutations for each exposure are in brackets next to label.

Previous studies have suggested the existence of exposure specific sets of sperm epimutations [12], so a comparison was made with a number of different exposure epimutation sets and the methoxychlor epimutations identified. A Venn diagram presented in Figure 9 indicates that the majority of methoxychlor intersection or average epimutations are unique with negligible overlap with the vinclozolin, pesticide (DEET and permethrin), DDT and plastics epimutations. No epimutations were found to be common between all the exposures. The highest overlap was observed between the plastic (BPA and phthalates) and pesticide (DEET and permethrin) as previously described [12], while for methoxychlor only 4 epimutations out of 37 intersection epimutations had any overlap (Figure 9). Therefore, the F3 generation transgenerational sperm epimutations induced by methoxychlor appear to be exposure specific.

Further analysis of the genes associated with the DMR in Table S7 demonstrate a number of functional gene pathways and cellular processes are potentially affected. Analysis of the association of the 311 average epimutations with functional signaling pathways and cellular processes used a KEGG pathway correlation as previously described [48]. The pathways with a statistically significant over-representation of epimutation associated genes is shown in Table 2. Therefore, the methoxychlor transgenerationally induced sperm epimutations have the potential to influence a variety of cellular pathways and processes.

**Table 2:** Pathway DMR associations.

Cellular Process or Pathway Name	Number of DMR Associated Genes	Number of Genes in Pathway	Fisher's Exact p-Value*
Olfactory transduction	6	15	3.24E-07
Steroid hormone biosynthesis	7	46	4.12E-05
Chemical carcinogenesis	6	35	0.000074
Ribosome	10	143	0.0007986
Drug metabolism - cytochrome P450	4	23	0.001175
Antigen processing and presentation	5	41	0.001508
Autoimmune thyroid disease	4	25	0.001625
Metabolic pathways	32	2435	0.00308
Phagosome	7	95	0.003587
Metabolism of xenobiotics by cytochrome P450	4	32	0.004123
Herpes simplex infection	8	126	0.004739
Graft-versus-host disease	3	17	0.004838
Endocytosis	8	139	0.008446
Allograft rejection	3	22	0.01015
Fatty acid elongation	3	22	0.01015
HTLV-I infection	10	205	0.01059
Type I diabetes mellitus	3	24	0.01294
Systemic lupus erythematosus	4	45	0.01385
Cell adhesion molecules (CAMs)	6	99	0.01699
Retinol metabolism	4	48	0.01724
Biosynthesis of unsaturated fatty acids	3	30	0.0237
Transcriptional misregulation in cancer	7	147	0.0336
Cardiac muscle contraction	4	60	0.03569
Viral myocarditis	3	38	0.04368
Phosphatidylinositol signaling system	3	39	0.04661
Wnt signaling pathway	5	98	0.05306

*Statistical likelihood of finding this many or more DMR associated genes in this pathway by Fisher's Exact Test.

## Discussion

The current study demonstrated the pesticide methoxychlor can promote the epigenetic transgenerational inheritance of disease following the exposure of an F0 generation gestating female. The mechanism of this non-genetic form of inheritance involves the transgenerational transmission of adult-onset disease susceptibility through epigenetic changes in the germline. Although obtaining sufficient numbers of eggs was not possible to investigate oocyte differential DNA methylation regions, epigenetic alterations in the sperm DNA of the F3 generation (great-grand offspring) were observed after methoxychlor exposure of the F0 generation gestating female ancestors. This transgenerational transmission of adult onset diseases has implications of disease risk for not only the individual exposed, but also for future generations.

The toxicological profile of methoxychlor in humans includes death, systemic (aplastic anemia), cardiovascular (low blood pressure), and neurological (blurred vision, dizziness and paresthesia) effects, as well as cancer (leukemia) [44]. Animal studies with methoxychlor documented adverse effects on fertility, early pregnancy and in utero development in females, and altered social behavior in males after prenatal exposure [13]. A recent toxicity study revealed a suppression of body weights, prolonged estrous cycles, reduced fertility, decreased numbers of implantation sites and newborns, decreased ovary weights, increased incidence of cystic ovary, increased uterine weights, delayed preputial separation, and reduced sperm counts and reproductive organ weights [16]. The current study examines the adult onset diseases in the direct exposed F1 generation fetus and in the transgenerational F3 generation offspring not exposed to methoxychlor. Although toxic methoxychlor doses were not used, the current study used intraperitoneal injection of a pharmacological dose based on 4% of the oral LD50 dose for methoxychlor. This dose is within the range of high environmental exposures [45]. No major toxic effects were observed in F1 generation animals. However, the dose and route of administration used do not allow risk assessment of methoxychlor exposure to be assessed. The objective of the study was to further investigate [2] if exposure to methoxychlor could promote the epigenetic transgenerational inheritance of disease phenotypes and not designed to do risk assessment of exposure to methoxychlor. Future work with appropriate modes of administration and doses can now use the current information to more efficiently do risk assessment. The current study does reveal the potential of methoxychlor to promote the epigenetic transgenerational transmission of disease.

In this study the epigenetic transgenerational inheritance of disease observed includes kidney disease, ovary disease and obesity. The transgenerational kidney disease was observed in male and female F3 generation descendents of F0 generation gestating females exposed to methoxychlor. The transgenerational kidney pathology was similar to that previously observed [7], [8], [9], [10], [11], [46] by other toxicants. In addition to the morphological defects, blood markers such as BUN alterations have previously been shown to support the presence of kidney disease [46]. Cystic tubular nephropathy has been found following methoxychlor exposure in mice [49]. Miniature swine given methoxychlor developed chronic renal disease in relatively short periods of time [26]. Proximal convoluted kidney tubules showed vacuolar degeneration changes after methoxychlor exposure [50] Kidney disease in the F3 generation offspring following methoxychlor exposure to F0 generation gestating females in the current study highlights the risk of transgenerational transmission of kidney disease.

Ovarian diseases observed included increased rates of primordial follicle pool reduction associated with primary ovarian insufficiency (POI) and polycystic ovarian disease (PCO). An increase in POI was found in the F1 generation and increased PCO was found in the F3 generations of methoxychlor lineage females. Methoxychlor's toxicity effects on the ovary have been demonstrated and reviewed [13], [16], [25], [35], [38], [40], [42], [51]. Worldwide, human female populations are facing an increase in primary ovarian insufficiency characterized by primordial follicle pool loss, and the most common reproductive disease in women is polycystic ovarian disease characterized by the presence of anovulatory cysts [52], [53]. These ovarian disease phenotypes, as shown in the current study, may be in part the outcome of ancestral exposures, such as methoxychlor, and epigenetic transgenerational inheritance. In the current study the POI disease was observed in the direct exposure F1 generation and the polycystic ovarian disease was found in the transgenerational F3 generation. In previous transgenerational exposure models with a variety of different toxicants the POI has been observed in F1 and F3 generations [7], [8], [9], [10], [11], [46], [48], while PCO was primarily observed in the F3 generation females [48]. Therefore, the absence of POI in the F3 generation methoxychlor lineage females was unique. The mechanism for the germline transmission of transgenerational disease involves a cell specific alteration in the epigenome and transcriptome of cells associated with disease. Previous observations have shown a transgenerational alteration in both the transcriptome and the epigenome of the ovarian granulosa cells from F3 generation vinclozolin lineage rats [48]. In addition, epigenetic mechanisms underlie the development of polycystic ovary syndrome in women [54] and prenatally androgenized rhesus monkeys [55]. Therefore, ancestral exposure to methoxychlor may contribute to the development of this ovarian disease through epigenetic transgenerational inheritance mechanisms. Observations suggest that this additional paradigm needs to be considered for the etiology of polycystic ovarian disease in women.

Methoxychlor was found to promote transgenerational obesity in approximately 25% of the females and 45% of the males of the F3 generation methoxychlor lineages in comparison to a 4% incidence in females and a 25% incidence in males in the F3 generation control lineage (Figure 3). Previously several environmental toxicants were found to promote the epigenetic transgenerational inheritance of obesity including plastics (BPA and phthalates) [7], hydrocarbons (jet fuel) [10], tributylin [56] and DDT [11]. In addition to environmental toxicants promoting transgenerational obesity, nutrition is also a factor in the occurrence of transgenerational obesity [57], [58], [59], [60]. Obesity is increasing in the United States population and elsewhere in the world. According to the Center for Disease Control in 2010, 33% of adults in the United States are obese and 17% of children between the ages of 2–19 are obese [61]. Obesity is a forerunner for many other diseases and the main adverse consequences of obesity are metabolic syndrome, cardiovascular disease, type 2 diabetes and a reduced average life expectancy [61], [62]. Obese women experience higher prevalence of amenorrhea, infertility and polycystic ovarian disease. Greater risks of pregnancy complications in these women such as hypertension, gestational diabetes and greater delivery problems occur, which may result in unplanned cesarean surgeries. Further, maternal obesity can have a negative effect on children's health [63]. Experimental studies in rats show that obese dams transmit obesity to the subsequent generation [64]. Waterland et al., (2008) [65] suggested that epigenetic mechanisms are involved in the transgenerational transmission of maternal obesity. Observations from the current study provide an example of an epigenetic transgenerational mechanism leading to an increase in obesity. The development of obesity as a result of ancestral exposure to environmental toxicants such as methoxychlor may be a component of the increase in obesity observed today. Future studies need to examine the adult status of obesity epigenetic markers, bone mineralization, adult bone length and metabolic disease in the F3 generation offspring of methoxychlor lineage to obtain insights into the pathogenesis of the adult-onset obesity observed. The suggestion that all these different disease phenotypes (kidney disease, ovary disease and obesity) may be linked to a complex disease syndrome that involves an environmentally induced epigenetic transgenerational inheritance mechanism needs to be considered as a potential component of the disease etiology.

Experiments were performed to determine the parental origin of the germline transmission of the transgenerational disease. Observations of the increased incidence of disease in the F4 generation (female) reverse outcross offspring, but not in the (male) outcross offspring demonstrated transgenerational transmission through the female germline. This is one of the first studies showing a transmission of increased incidence of kidney disease in females and males, and obesity in males through the female germline after toxicant exposure to pregnant F0 generation females. Previous studies with vinclozolin [2] or high fat diet [57] showed transmission of increased incidence of disease through the male germline. Recently, DDT induced epigenetic transgenerational inheritance of obesity and other disease was found to involve a combination of male and female germline transmission transgenerationally [11]. Interestingly, for DDT the male obesity was transmitted through the female germline and female obesity transmitted through the male germline [11]. In the current study, methoxychlor male obesity was found to be transmitted through the female germline (Figure 5), but the female obesity was not observed in either the male or female germline transmission alone. Therefore, the transmission of transgenerational female obesity may involve a combination of male and female germline transmission. Previously, the optimal transgenerational transmission of disease appeared to involve a combined contribution of the male and female germline transmission [3], [7], [8], [9], [10], [11]. The current study demonstrates that female germline transmission of environmentally induced epigenetic transgenerational phenotypes is equally as stable as male germline transmission. Future studies will need to elucidate the sex specific roles and mechanisms involved.

One of the established mechanisms for the epigenetic transgenerational inheritance of disease and phenotypic variation is the reprogramming of the germline epigenome during gonadal sex determination [1], [66]. The germline epigenome (DNA methylation) is altered during gonadal development [67] and during gametogenesis appears to be permanently reprogrammed like an imprinted site and to be protected from DNA demethylation and reprogramming at fertilization in the following generations. The parent of origin of allelic transmission and differential DNA methylation programming suggest these epimutations are imprinted-like, but mono-allelic gene expression remains to be investigated. The transgenerational transmission of the modified germline epimutation results in modification of all somatic cell and tissue epigenomes and transcriptomes, promoting disease susceptibility and the epigenetic transgenerational inheritance of disease phenotypes [1]. The current study investigated the transgenerational sperm epigenome and presence of epimutations induced in the methoxychlor lineage animals. The epigenetic analysis of the female germline was not possible due to the limited number of eggs available. Future studies will need to investigate the female germline, however, the current study focused on the male germline. The current study identified the differentially DNA methylated regions (DMR), defined as epimutations, in the methoxychlor lineage F3 generation sperm as previously described [12]. A total of 37 intersection epimutations that were reproducible in all three experiments and 311 average epimutations were identified and associated with gene promoters. These epimutations were induced by the ancestral exposure to methoxychlor. The list of the DMR from methoxychlor lineage F3 generation sperm is presented in Table 1 and Table S7. The chromosomal locations of the transgenerational epimutations demonstrated that all chromosomes had epimutations and a number of the average epimutations were statistically over-represented in clusters of epimutations in regions of a two megabase size. These regions may be more susceptible to acquiring epimutations due to specific genomic features as previously described [4], [6]. Analysis of the genes associated with these epimutations demonstrated a wide variety of different functional gene pathways potentially effected have been previously correlated to disease (Table 2). Therefore, the observations support the presence of transgenerational sperm epimutations that will in part mediate the transgenerational inheritance of disease observed. Future studies will need to identify and compare the female germline epigenome modifications.

Environmentally induced epigenetic transgenerational inheritance of disease requires the absence of any direct exposure or genetic manipulation [1]. Therefore, for gestating female exposures the F1 generation involves direct exposure and the F3 generation is the first transgenerational generation with no exposure [5]. Environmentally induced pathologies such as uterine defects can promote pathologies in the offspring of the environmentally exposed individuals, as seen with diethylstilbesterol (DES) [68]. However, in the absence of any environmental exposure the only mechanism to transmit information from one generation to the next is through the germline. In the case of epigenetic transgenerational inheritance this non-genetic form of inheritance transmits epimutations as observed in the current study. Although the later life adult onset disease phenotype may be secondarily derived from an early life abnormality such as uterine pathology or metabolic defects, the initial causal molecular event for the transgenerational phenotype is the germline epigenetic alteration. An experiment with spermatogenic cell germline transplantation in a phthalate lineage transgenerational model [69] supports this requirement for the germline epigenetic modification for the transgenerational phenotype.

A variety of different environmental toxicants have been shown to induce the epigenetic transgenerational inheritance of disease and abnormal phenotypes [2], [3], [4], [7], [8], [9], [10], [11], [12], [56], [69], [70]. For example, gestational exposure to vinclozolin resulted in F3 generation disease including testis, prostate and kidney disease, immune system abnormalities, tumors, uterine hemorrhage during pregnancy, and polycystic ovarian disease [2], [3], [46], [47]. In addition, alterations in the methylation patterns of imprinted genes in sperm of the F3 generation male mice were found after vinclozolin exposure [70]. Exposure of F0 generation gestating rats to Bisphenol-A resulted in reduced fertility in F3 generation males [71]. Additional environmental factors such as nutrition [65], [72] and stress [73] also can promote epigenetic transgenerational inheritance of disease phenotypes. Environmentally induced epigenetic transgenerational inheritance has been demonstrated in worms [74], flies [75], plants [76], fish [77], rodents [2] and mammals [78], [79], [80] indicating this phenomenon will likely be important in general biology and disease etiology [1]. Observations demonstrate that exposure of gestating females to methoxychlor during gonadal sex determination promotes epigenetic transgenerational inheritance of adult-onset disease. These disease phenotypes have an impact on fertility and reproduction. The overall increase in total transgenerational disease and multiple disease are considerable. Interestingly, the increased disease incidence in F4 generation reverse (female) outcross offspring indicated that the transgenerational disease transmission was primarily through the maternal germline. Associated with the incidence of these transgenerational diseases are transgenerational epigenetic epimutations in the sperm DNA, while female germline epigenetic effects remain to be elucidated. These epimutations may be useful as early stage biomarkers of compound exposure and adult onset disease. Although not designed for risk assessment, these results have implications for the human population that is exposed to methoxychlor that may experience declines in fertility and increases in adult onset diseases with a potential to transmit these to subsequent generations.

## Materials and Methods

### Animal studies and breeding

Female and male rats of an outbred strain Hsd:Sprague Dawley, SD (Harlan) at about 70 and 100 days of age were fed ad lib with a standard rat diet and ad lib tap water for drinking. To obtain time-pregnant females, the female rats in proestrus were pair-mated with male rats. The sperm-positive (day 0) rats were monitored for diestrus and body weight. On days 8 through 14 of gestation [47], the females were administered daily intraperitoneal injections of methoxychlor (200 mg/kg BW/day) or dimethyl sulfoxide (vehicle). Treatment lineages are designated control or methoxychlor lineages. The gestating female rats treated were designated as the F0 generation. The offspring of the F0 generation rats were the F1 generation. Non-littermate females and males aged 70–90 days from F1 generation of control or methoxychlor lineages were bred to obtain F2 generation offspring. The F2 generation rats were bred to obtain F3 generation offspring. Outcross F4 generation offspring (n = 8 litters per lineage) were obtained by breeding the F3 generation males from control and methoxychlor lineages with wild type females. Reverse outcross F4 generation progeny (n = 8 litters per lineage) were obtained by breeding the F3 generation females from control and methoxychlor lineages with wild type males. The outcross and the reverse outcross individuals were maintained until 10 months of age and then euthanized for tissue collection and disease evaluation. Onset of puberty was assessed in females by daily examination for vaginal opening from 30 days of age and in males by balano-preputial separation from 35 days of age. The F1–F3 generation offspring were not exposed directly to the methoxychlor treatment. All experimental protocols for the procedures with rats were pre-approved by the Washington State University Animal Care and Use Committee (IACUC approval # 02568-031).

### Tissue harvest and histology processing

Rats at 10–12 months of age were euthanized by CO_2_ inhalation for tissue harvest. Body and organ weights were measured at dissection time. Testis, epididymis, prostate, seminal vesicle, ovaries, uterus and kidney were fixed in Bouin's solution (Sigma) and 70% ethanol, then processed for paraffin embedding by standard procedures for histopathology examination. Five-micrometer tissue sections were made and were either unstained and used for TUNEL analysis or stained with H & E stain and examined for histopathology. Blood samples were collected at the time of dissection, allowed to clot, centrifuged and serum samples stored at −20°C for steroid hormone assays.

### Testicular apoptotic cells by TUNEL

Testis sections were examined by Terminal deoxynucleotidyl transferase-mediated dUTP nick end labeling (TUNEL) assay (In situ cell death detection kit, Fluorescein, Roche Diagnostics, Mannheim, Germany). Sections were deparaffinized and rehydrated through an alcohol series. They were deproteinized by Proteinase K (20 mg/ml; Invitrogen, Carlsbad, CA), washed with PBS and then 25 µl of the enzyme-label solution mix was applied to the sections and incubated at 37°C for 90 min. After PBS washes, slides were mounted and kept at 4°C until examination in a fluorescent microscope in dark field. Both testis sections of each slide were microscopically examined to identify and to count apoptotic germ cells by their bright fluorescence.

### Histopathology examination and disease classification

Testis histopathology criteria included the presence of a vacuole, azoospermic atretic seminiferous tubule and ‘other’ abnormalities including sloughed spermatogenic cells in center of the tubule and a lack of a tubule lumen. Prostate histopathology criteria included the presence of vacuoles, atrophic epithelial layer of ducts and hyperplasia of prostatic duct epithelium. Kidney histopathology criteria included reduced size of glomerulus, thickened Bowman's capsule and the presence of proteinaceous fluid-filled cysts. A cut-off was established to declare a tissue ‘diseased’ based on the mean number of histopathological abnormalities plus two standard deviations from the mean of control tissues by each of the three individual observers scoring the tissues. This number was used to classify rats into those with and without testis, prostate or kidney disease in each lineage. A rat tissue section was finally declared ‘diseased’ only when at least two of the three observers marked the same tissue section ‘diseased’. The occurrence of obesity was determined by statistically significant weight gain and excessive accumulation of subcutaneous and intra-abdominal adipose tissue. The proportion of rats with obesity, uterine disease or tumor development was obtained by accounting those that had these conditions out of all the animals evaluated.

### Ovarian disease analysis by follicle and cyst counts

Every 30^th^ section of each pair of ovaries was stained with hematoxylin and eosin and three stained sections (150 µm apart) through the central portion of the ovary with the largest cross section were evaluated. Ovary sections were assessed for two diseases: primordial follicle loss and polycystic ovary disease. Primordial follicle loss was determined by counting the number of primordial follicles per ovary section and averaging across three sections. An animal was scored as having primordial follicle loss if the primordial follicle number was less than that of the control mean minus two standard deviations. Primordial follicles had an oocyte surrounded by a single layer of either squamous or both squamous and cuboidal granulosa cells [81], [82]. Follicles had to be non-atretic and showing an oocyte nucleus in order to be counted. Polycystic ovary was determined by microscopically counting the number of small cystic structures per section averaged across three sections. A polycystic ovary was defined as having a number of small cysts that was more than the control mean plus two standard deviations. Cysts were defined as fluid-filled structures of a specified size that were not filled with red blood cells and which were not follicular antra. A single layer of cells may line cysts. Small cysts were 50 to 250 µm in diameter measured from the inner cellular boundary across the longest axis. Percentages of females with primordial follicle loss or polycystic ovarian disease were computed.

### Analysis of puberty onset

For identifying a rat with a pubertal abnormality the mean from all the rats in control lineage evaluated for pubertal onset was computed and its standard deviation calculated. A range of normal pubertal onset was chosen based on the mean ±2 standard deviations. Any rat with a pubertal onset below this range was considered to have had an early pubertal onset and any rat with a pubertal onset above this range was considered to have had a delayed pubertal onset. The proportion of rats with pubertal abnormalities was computed from the total number of rats evaluated.

### Overall disease incidence

A table of the incidence of individual diseases in rats from each lineage was created and the proportion of individual disease, total disease and multiple disease incidences was computed. For the individual diseases, only those rats that showed a presence of disease (plus) or absence of disease (minus) are included in the computation. For the total diseases, a column with total number of diseases for each rat was created and the number of plus signs were added up for each of the rats and the proportion was computed as the numbers of rats with total disease out of all the listed rats. For the multiple diseases, the proportion was computed as the number of rats with multiple disease out of all the listed rats.

### Epididymal sperm collection, DNA isolation and methylated DNA immunoprecipitation (MeDIP)

The epididymis was dissected free of connective tissue, a small cut made to the cauda and placed in 5 ml of F12 culture medium containing 0.1% bovine serum albumin for 10 minutes at 37°C and then kept at 4°C to immobilize the sperm. The epididymal tissue was minced and the released sperm centrifuged at 13,000×*g* and stored in fresh NIM buffer at −20°C until processed further. Sperm heads were separated from tails through sonication following previously described protocol (without protease inhibitors) [83] and then purified using a series of washes and centrifugations [84] from a total of nine F3 generation rats per lineage (control or methoxychlor) that were 120 days of age. DNA extraction on the purified sperm heads was performed as described [4]. Equal concentrations of DNA from three individual sperm samples were used to produce three DNA pools per lineage and employed for chromatin immunoprecipitation of methylated DNA fragments (MeDIP). MeDIP was performed as previously described [4], [12].

### MeDIP-Chip Analysis

The comparative MeDIP-Chip was performed with Roche Nimblegen's Rat DNA Methylation 3×720K CpG Island Plus RefSeq Promoter Array which contains three identical sub-arrays, with 720,000 probes per sub-array, scanning a total of 15,287 promoters (3,880 bp upstream and 970 bp downstream from transcription start site). Probe sizes range from 50–75 bp in length with the median probe spacing of 100 bp. Three different comparative (MeDIP vs. MeDIP) hybridization experiments were performed (3 sub-arrays) for methoxychlor lineage versus control, with each subarray encompassing DNA samples from 6 animals (3 each from methoxychlor and control). MeDIP DNA samples from experimental lineages were labeled with Cy3 and MeDIP DNA samples from the control lineage were labeled with Cy5.

### Bioinformatic and Statistical Analyses of Chip Data

All the MeDIP-Chip raw hybridization data has been deposited in the NCBI GEO database (GEO # GSE58091) and is also available, along with the R-Code, used at www.skinner.wsu.edu. The data was imported into R (R Development Core Team (2010), R: A language for statistical computing, R Foundation for Statistical Computing. Vienna, Austria. ISBN 3-900051-07-0. URL http//www.R-project.org) and then annotation packages were built using pdInfoBuilder (Seth Falcon and Benilton Carvalho pdInfoBuilder: Platform Design Information Package Builder, R version 1.24.0) for platform design information. The oligo package [85] was used to read in the Nimblegen (XYS) files. The bioinformatic analysis was performed as previously described [4], [12]. The statistical analysis was performed in pairs of comparative IP hybridizations between methoxychlor (M) and controls (C) (e.g. M1-C1 and M2-C2; M1-C1 and M3-C3; M2-C2 and M3-C3). In order to assure the reproducibility of the candidates obtained, the intersection epimutations candidates showing significant changes in all of the single paired comparisons were examined as a having a significant change in DNA methylation between methoxychlor lineage and control lineage. This is a very stringent approach to select for changes, since it only considers repeated changes in all paired analysis. Alternately, the average epimutations obtained through an average of the three experiments (i.e. comparisons) were also examined. Clustered regions of interest were then determined by combining consecutive probes within 600 bases of each other, and based on whether their mean M values were positive or negative, with significance P-values less than 10^−5^. The statistically significant differential DNA methylated regions were identified and P-value associated with each region presented. Each region of interest was then annotated for gene and CpG content. This list was further reduced to those regions with an average intensity value exceeding 9.5 (log scale) and a CpG density ≥1 CpG/100 bp.

### Statistical analysis of rat organ and disease data

For statistical analysis, all of the data on body and organ weights were used as input in the program GraphPad© Prism 5 statistical analysis program and t-tests were used to determine if the data from the methoxychlor lineage differed from those of control lineages. For the number of rats with disease, logistic regression analysis was used to analyze the data (control or methoxychlor and diseased or unaffected). All treatment differences were considered significant if P value was less than 0.05.

## Supporting Information

Figure S1 **Transgenerational animal hormone levels.** A. Serum estradiol concentrations in proestrus-estrus in F3 generation control and methoxychlor lineage females. B. Serum estradiol concentrations in diestrus in F3 generation control and methoxychlor lineage females. C. Serum testosterone concentrations in the F3 generation control and methoxychlor lineage males.
(PDF)

Figure S2 **Ancestral exposure to methoxychlor and adult-onset transgenerational testis disease**. Percentages of the F1 and F3 generation males of control and methoxychlor lineages with testis disease (panel A) or prostate disease (panel B). The number of diseased rats / total number of rats is shown above the respective bar graphs. Micrographs (scale bar  = 100 µm) show testis disease (control: panel C; methoxychlor: panel E) and prostate disease (control: panel D; methoxychlor: panel F) in F3 generation rats.
(PDF)

Figure S3 **Testicular spermatogenic cell apoptosis.** Number of apoptotic germ cells in F1 and F3 generation control (open bars) and methoxychlor (black bars) lineages evaluated by TUNEL assay.
(PDF)

Table S1 (A) Body Weight and organ weights in F1 and F3 generation female rats of Control and Methoxychlor lineages (mean ± standard error). Asterisks (*, **, ***), if present, indicate statistically significant differences between means of Control and Methoxychlor lineages (P<0.05, P<0.01 and P<0.001 respectively); nd  =  not determined. (B) Body weight (grams) and organ weights (% of body weight) in F1 and F3 generation male rats of Control and Methoxychlor lineages (mean ± standard error). Asterisks (*, **), if present, indicate statistically significant differences between means of Control and Methoxychlor lineages (P<0.05, P<0.01 respectively); nd  =  not determined.
(PDF)

Table S2 (A) Individual disease incidence in F1 generation female rats of Control and Methoxychlor lineages. (B) Individual disease incidence in F1 generation male rats of Control and Methoxychlor lineages.
(PDF)

Table S3 (A) Individual disease incidence in F3 generation female rats of Control and Methoxychlor lineages. (B) Individual disease incidence in F3 generation male rats of Control and Methoxychlor lineages.
(PDF)

Table S4 (A) Body Weight in F4 generation Outcross and Reverse Outcross female rats of Control and Methoxychlor lineages (mean ± standard error). (B) Body weight (grams) in F4 generation Outcross and Reverse Outcross male rats of Control and Methoxychlor lineages (mean ± standard error).
(PDF)

Table S5 (A) Individual disease incidence in F4 generation Outcross female rats of Control and Methoxychlor lineages. (B) Individual disease incidence in F4 generation Outcross male rats of Control and Methoxychlor lineages.
(PDF)

Table S6 (A) Individual disease incidence in F4 generation Reverse Outcross female rats of Control and Methoxychlor lineages. (B) Individual disease incidence in F4 generation Reverse Outcross male rats of Control and Methoxychlor lineages.
(PDF)

Table S7 **Methoxychlor lineage F3 generation sperm average epimutations.**
(PDF)

Table S8 **Characteristics of the average epimutation clusters and associated genes.** Clusters with the same gene listed more than once indicates multiple epimutations associated with that gene.
(PDF)

## References

[1] Skinner MK, Manikkam M, Guerrero-Bosagna C (2010) Epigenetic transgenerational actions of environmental factors in disease etiology. Trends Endocrinol Metab 21: 214–222. 20074974 10.1016/j.tem.2009.12.007 PMC2848884

[2] Anway MD, Cupp AS, Uzumcu M, Skinner MK (2005) Epigenetic transgenerational actions of endocrine disruptors and male fertility. Science 308: 1466–1469. 15933200 10.1126/science.1108190 PMC11423801

[3] Guerrero-Bosagna C, Covert T, Haque MM, Settles M, Nilsson EE, et al (2012) Epigenetic Transgenerational Inheritance of Vinclozolin Induced Mouse Adult Onset Disease and Associated Sperm Epigenome Biomarkers. Reproductive Toxicology 34: 694–707. 23041264 10.1016/j.reprotox.2012.09.005 PMC3513496

[4] Guerrero-Bosagna C, Settles M, Lucker B, Skinner M (2010) Epigenetic transgenerational actions of vinclozolin on promoter regions of the sperm epigenome. PLoS ONE 5: e13100. 20927350 10.1371/journal.pone.0013100 PMC2948035

[5] Skinner MK (2011) Environmental epigenetic transgenerational inheritance and somatic epigenetic mitotic stability. Epigenetics 6: 838–842. 21637037 10.4161/epi.6.7.16537 PMC5703187

[6] Skinner MK, Manikkam M, Haque MM, Zhang B, Savenkova M (2012) Epigenetic Transgenerational Inheritance of Somatic Transcriptomes and Epigenetic Control Regions. Genome Biol 13: R91. 23034163 10.1186/gb-2012-13-10-r91 PMC3491419

[7] Manikkam M, Tracey R, Guerrero-Bosagna C, Skinner M (2013) Plastics Derived Endocrine Disruptors (BPA, DEHP and DBP) Induce Epigenetic Transgenerational Inheritance of Adult-Onset Disease and Sperm Epimutations. PLoS ONE 8: e55387. 23359474 10.1371/journal.pone.0055387 PMC3554682

[8] Manikkam M, Tracey R, Guerrero-Bosagna C, Skinner M (2012) Pesticide and Insect Repellent Mixture (Permethrin and DEET) Induces Epigenetic Transgenerational Inheritance of Disease and Sperm Epimutations. Reproductive Toxicology 34: 708–719. 22975477 10.1016/j.reprotox.2012.08.010 PMC3513590

[9] Manikkam M, Tracey R, Guerrero-Bosagna C, Skinner MK (2012) Dioxin (TCDD) induces epigenetic transgenerational inheritance of adult onset disease and sperm epimutations. PLoS ONE 7: e46249. 23049995 10.1371/journal.pone.0046249 PMC3458876

[10] Tracey R, Manikkam M, Guerrero-Bosagna C, Skinner M (2013) Hydrocarbon (Jet Fuel JP-8) Induces Epigenetic Transgenerational Inheritance of Adult-Onset Disease and Sperm Epimutations. Reproductive Toxicology 36: 104–116. 23453003 10.1016/j.reprotox.2012.11.011 PMC3587983

[11] Skinner MK, Manikkam M, Tracey R, Nilsson E, Haque MM, et al (2013) Ancestral DDT Exposures Promote Epigenetic Transgenerational Inheritance of Obesity BMC Medicine. 11: 228. 10.1186/1741-7015-11-228 PMC3853586 24228800

[12] Manikkam M, Guerrero-Bosagna C, Tracey R, Haque MM, Skinner MK (2012) Transgenerational Actions of Environmental Compounds on Reproductive Disease and Epigenetic Biomarkers of Ancestral Exposures. PLoS ONE 7: e31901. 22389676 10.1371/journal.pone.0031901 PMC3289630

[13] Cummings AM (1997) Methoxychlor as a model for environmental estrogens. Crit Rev Toxicol 27: 367–379. 9263644 10.3109/10408449709089899

[14] Methoxychlor. IARC Monogr Eval Carcinog Risk Chem Hum 20: 259–281. 397167

[15] Duggan RE, Corneliussen PE, Duggan MB, McMahon BM, Martin RJ (1983) Pesticide residue levels in foods in the United States from July 1, 1969, to June 30, 1976: Summary. J Assoc Off Anal Chem 66: 1534–1535. 6643370

[16] Aoyama H, Hojo H, Takahashi KL, Shimizu-Endo N, Araki M, et al (2012) Two-generation reproduction toxicity study in rats with methoxychlor. Congenit Anom (Kyoto) 52: 28–41. 22348781 10.1111/j.1741-4520.2011.00344.x

[17] Akingbemi BT, Ge RS, Klinefelter GR, Gunsalus GL, Hardy MP (2000) A metabolite of methoxychlor, 2,2-bis(p-hydroxyphenyl)-1,1, 1-trichloroethane, reduces testosterone biosynthesis in rat leydig cells through suppression of steady-state messenger ribonucleic acid levels of the cholesterol side-chain cleavage enzyme. Biol Reprod 62: 571–578. 10684797 10.1095/biolreprod62.3.571

[18] Murono EP, Derk RC (2005) The reported active metabolite of methoxychlor, 2,2-bis(p-hydroxyphenyl)-1,1,1-trichloroethane, inhibits testosterone formation by cultured Leydig cells from neonatal rats. Reprod Toxicol 20: 503–513. 16199348 10.1016/j.reprotox.2005.03.002

[19] Murono EP, Derk RC, Akgul Y (2006) In vivo exposure of young adult male rats to methoxychlor reduces serum testosterone levels and ex vivo Leydig cell testosterone formation and cholesterol side-chain cleavage activity. Reprod Toxicol 21: 148–153. 16226009 10.1016/j.reprotox.2005.08.005

[20] Amstislavsky SY, Amstislavskaya TG, Amstislavsky VS, Tibeikina MA, Osipov KV, et al (2006) Reproductive abnormalities in adult male mice following preimplantation exposures to estradiol or pesticide methoxychlor. Reprod Toxicol 21: 154–159. 16162399 10.1016/j.reprotox.2005.07.009

[21] Cupp AS, Skinner MK (2001) Actions of the endocrine disruptor methoxychlor and its estrogenic metabolite on in vitro embryonic rat seminiferous cord formation and perinatal testis growth. Reprod Toxicol 15: 317–326. 11390175 10.1016/s0890-6238(01)00124-1

[22] Cupp AS, Uzumcu M, Suzuki H, Dirks K, Phillips B, et al (2003) Effect of transient embryonic in vivo exposure to the endocrine disruptor methoxychlor on embryonic and postnatal testis development. J Androl 24: 736–745. 12954667 10.1002/j.1939-4640.2003.tb02736.x

[23] Wang XJ, Bartolucci-Page E, Fenton SE, You L (2006) Altered mammary gland development in male rats exposed to genistein and methoxychlor. Toxicol Sci 91: 93–103. 16443925 10.1093/toxsci/kfj120

[24] You L, Sar M, Bartolucci EJ, McIntyre BS, Sriperumbudur R (2002) Modulation of mammary gland development in prepubertal male rats exposed to genistein and methoxychlor. Toxicol Sci 66: 216–225. 11896288 10.1093/toxsci/66.2.216

[25] Anway MD, Skinner MK (2006) Epigenetic transgenerational actions of endocrine disruptors. Endocrinology 147: S43–49. 16690803 10.1210/en.2005-1058

[26] Reuber MD (1980) Carcinogenicity and toxicity of methoxychlor. Environ Health Perspect 36: 205–219. 7000513 10.1289/ehp.8036205 PMC1637742

[27] Swartz WJ, Eroschenko VP (1998) Neonatal exposure to technical methoxychlor alters pregnancy outcome in female mice. Reprod Toxicol 12: 565–573. 9875691 10.1016/s0890-6238(98)00041-0

[28] Alworth LC, Howdeshell KL, Ruhlen RL, Day JK, Lubahn DB, et al (2002) Uterine responsiveness to estradiol and DNA methylation are altered by fetal exposure to diethylstilbestrol and methoxychlor in CD-1 mice: effects of low versus high doses. Toxicol Appl Pharmacol 183: 10–22. 12217638 10.1006/taap.2002.9459

[29] Eroschenko VP, Abuel-Atta AA, Grober MS (1995) Neonatal exposures to technical methoxychlor alters ovaries in adult mice. Reprod Toxicol 9: 379–387. 7496094 10.1016/0890-6238(95)00025-6

[30] Borgeest C, Symonds D, Mayer LP, Hoyer PB, Flaws JA (2002) Methoxychlor may cause ovarian follicular atresia and proliferation of the ovarian epithelium in the mouse. Toxicol Sci 68: 473–478. 12151644 10.1093/toxsci/68.2.473

[31] Palanza P, Morellini F, Parmigiani S, vom Saal FS (1999) Prenatal exposure to endocrine disrupting chemicals: effects on behavioral development. Neurosci Biobehav Rev 23: 1011–1027. 10580314 10.1016/s0149-7634(99)00033-0

[32] Stoker TE, Robinette CL, Cooper RL (1999) Perinatal exposure to estrogenic compounds and the subsequent effects on the prostate of the adult rat: evaluation of inflammation in the ventral and lateral lobes. Reprod Toxicol 13: 463–472. 10613394 10.1016/s0890-6238(99)00049-0

[33] Takeuchi Y, Kosaka T, Hayashi K, Takeda M, Yoshida T, et al (2002) Thymic atrophy induced by methoxychlor in rat pups. Toxicol Lett 135: 199–207. 12270678 10.1016/s0378-4274(02)00259-x

[34] Latchoumycandane C, Mathur PP (2002) Effect of methoxychlor on the antioxidant system in mitochondrial and microsome-rich fractions of rat testis. Toxicology 176: 67–75. 12062931 10.1016/s0300-483x(02)00138-5

[35] Armenti AE, Zama AM, Passantino L, Uzumcu M (2008) Developmental methoxychlor exposure affects multiple reproductive parameters and ovarian folliculogenesis and gene expression in adult rats. Toxicol Appl Pharmacol 233: 286–296. 18848953 10.1016/j.taap.2008.09.010 PMC2613954

[36] Eroschenko VP, Amstislavsky SY, Schwabel H, Ingermann RL (2002) Altered behaviors in male mice, male quail, and salamander larvae following early exposures to the estrogenic pesticide methoxychlor. Neurotoxicol Teratol 24: 29–36. 11836069 10.1016/s0892-0362(01)00194-5

[37] Bertolasio J, Fyfe S, Snyder BW, Davis AM (2011) Neonatal injections of methoxychlor decrease adult rat female reproductive behavior. Neurotoxicology 32: 809–813. 21726579 10.1016/j.neuro.2011.06.007

[38] Gore AC, Walker DM, Zama AM, Armenti AE, Uzumcu M (2011) Early life exposure to endocrine-disrupting chemicals causes lifelong molecular reprogramming of the hypothalamus and premature reproductive aging. Mol Endocrinol 25: 2157–2168. 22016562 10.1210/me.2011-1210 PMC3231835

[39] Mahoney MM, Padmanabhan V (2010) Developmental programming: impact of fetal exposure to endocrine-disrupting chemicals on gonadotropin-releasing hormone and estrogen receptor mRNA in sheep hypothalamus. Toxicol Appl Pharmacol 247: 98–104. 20621667 10.1016/j.taap.2010.05.017 PMC2914852

[40] Zama AM, Uzumcu M (2009) Fetal and neonatal exposure to the endocrine disruptor methoxychlor causes epigenetic alterations in adult ovarian genes. Endocrinology 150: 4681–4691. 19589859 10.1210/en.2009-0499 PMC2754680

[41] Stouder C, Paoloni-Giacobino A (2011) Specific transgenerational imprinting effects of the endocrine disruptor methoxychlor on male gametes. Reproduction 141: 207–216. 21062904 10.1530/REP-10-0400

[42] Uzumcu M, Zama AM, Oruc E (2012) Epigenetic mechanisms in the actions of endocrine-disrupting chemicals: gonadal effects and role in female reproduction. Reprod Domest Anim 47 Suppl 4338–347. 22827390 10.1111/j.1439-0531.2012.02096.x PMC4151320

[43] Zama AM, Uzumcu M (2013) Targeted genome-wide methylation and gene expression analyses reveal signaling pathways involved in ovarian dysfunction after developmental EDC exposure in rats. Biol Reprod 88: 52. 23303685 10.1095/biolreprod.112.104802 PMC3589238

[44] ATSDR. (2002) Toxicological profile for methoxychlor. U.S. Department of Health and Human Services, Public Health Service, Agency for Toxic Substances and Disease Registry. ATSDR. 38252767

[45] Palanza P, Morellini F, Parmigiani S, vom Saal FS (2002) Ethological methods to study the effects of maternal exposure to estrogenic endocrine disrupters: a study with methoxychlor. Neurotoxicol Teratol 24: 55–69. 11836072 10.1016/s0892-0362(01)00191-x

[46] Anway MD, Leathers C, Skinner MK (2006) Endocrine disruptor vinclozolin induced epigenetic transgenerational adult-onset disease. Endocrinology 147: 5515–5523. 16973726 10.1210/en.2006-0640 PMC5940332

[47] Nilsson EE, Anway MD, Stanfield J, Skinner MK (2008) Transgenerational epigenetic effects of the endocrine disruptor vinclozolin on pregnancies and female adult onset disease. Reproduction 135: 713–721. 18304984 10.1530/REP-07-0542 PMC5703189

[48] Nilsson E, Larsen G, Manikkam M, Guerrero-Bosagna C, Savenkova M, et al (2012) Environmentally Induced Epigenetic Transgenerational Inheritance of Ovarian Disease. PLoS ONE 7: e36129. 22570695 10.1371/journal.pone.0036129 PMC3343040

[49] Tullner WW, Edgcomb JH (1962) Cystic tubular nephropathy and decrease in testicular weight in rats following oral methoxychlor treatment. J Pharmacol Exp Ther 138: 126–130. 13994738

[50] Zaleska-Freljan KI, Kosicka B, Zbiegieni B (1983) The histological changes in some organs of the laboratory mice after intragastrically given bromfenvinphos and mixture of bromfenvinphos with methoxychlor. Pol J Pharmacol Pharm 35: 185–193. 6622299

[51] Craig ZR, Wang W, Flaws JA (2011) Endocrine-disrupting chemicals in ovarian function: effects on steroidogenesis, metabolism and nuclear receptor signaling. Reproduction 142: 633–646. 21862696 10.1530/REP-11-0136

[52] Vujovic S (2009) Aetiology of premature ovarian failure. Menopause Int 15: 72–75. 19465673 10.1258/mi.2009.009020

[53] Hart R, Hickey M, Franks S (2004) Definitions, prevalence and symptoms of polycystic ovaries and polycystic ovary syndrome. Best Pract Res Clin Obstet Gynaecol 18: 671–683. 15380140 10.1016/j.bpobgyn.2004.05.001

[54] Xu N, Azziz R, Goodarzi MO (2010) Epigenetics in polycystic ovary syndrome: a pilot study of global DNA methylation. Fertil Steril 94: : 781–783 e781. 10.1016/j.fertnstert.2009.10.020 PMC2889203 19939367

[55] Xu N, Kwon S, Abbott DH, Geller DH, Dumesic DA, et al (2011) Epigenetic mechanism underlying the development of polycystic ovary syndrome (PCOS)-like phenotypes in prenatally androgenized rhesus monkeys. PLoS ONE 6: e27286. 22076147 10.1371/journal.pone.0027286 PMC3208630

[56] Chamorro-Garcia R, Sahu M, Abbey RJ, Laude J, Pham N, et al (2013) Transgenerational inheritance of increased fat depot size, stem cell reprogramming, and hepatic steatosis elicited by prenatal exposure to the obesogen tributyltin in mice. Environ Health Perspect 121: 359–366. 23322813 10.1289/ehp.1205701 PMC3621201

[57] Dunn GA, Bale TL (2011) Maternal high-fat diet effects on third-generation female body size via the paternal lineage. Endocrinology 152: 2228–2236. 21447631 10.1210/en.2010-1461 PMC3100614

[58] Li J, Huang J, Li JS, Chen H, Huang K, et al (2012) Accumulation of endoplasmic reticulum stress and lipogenesis in the liver through generational effects of high fat diets. J Hepatol 56: 900–907. 22173165 10.1016/j.jhep.2011.10.018

[59] Massiera F, Barbry P, Guesnet P, Joly A, Luquet S, et al (2010) A Western-like fat diet is sufficient to induce a gradual enhancement in fat mass over generations. J Lipid Res 51: 2352–2361. 20410018 10.1194/jlr.M006866 PMC2903802

[60] Pentinat T, Ramon-Krauel M, Cebria J, Diaz R, Jimenez-Chillaron JC (2010) Transgenerational inheritance of glucose intolerance in a mouse model of neonatal overnutrition. Endocrinology 151: 5617–5623. 20943806 10.1210/en.2010-0684

[61] CDC. (2010) Division of Nutrition, Physical Activity, and Obesity, National Center for Chronic Disease Prevention and Health Promotion.

[62] McMillen IC, Rattanatray L, Duffield JA, Morrison JL, MacLaughlin SM, et al (2009) The early origins of later obesity: pathways and mechanisms. Adv Exp Med Biol 646: 71–81. 19536665 10.1007/978-1-4020-9173-5_8

[63] Linne Y (2004) Effects of obesity on women's reproduction and complications during pregnancy. Obes Rev 5: 137–143. 15245382 10.1111/j.1467-789X.2004.00147.x

[64] Campos KE, Volpato GT, Calderon IM, Rudge MV, Damasceno DC (2008) Effect of obesity on rat reproduction and on the development of their adult offspring. Braz J Med Biol Res 41: 122–125. 18235969 10.1590/s0100-879x2008005000001

[65] Waterland RA, Travisano M, Tahiliani KG, Rached MT, Mirza S (2008) Methyl donor supplementation prevents transgenerational amplification of obesity. Int J Obes (Lond) 32: 1373–1379. 18626486 10.1038/ijo.2008.100 PMC2574783

[66] Jirtle RL, Skinner MK (2007) Environmental epigenomics and disease susceptibility. Nat Rev Genet 8: 253–262. 17363974 10.1038/nrg2045 PMC5940010

[67] Skinner M, Guerrero-Bosagna C, Haque MM, Nilsson E, Bhandari R, et al (2013) Environmentally Induced Transgenerational Epigenetic Reprogramming of Primordial Germ Cells and Subsequent Germline. PLoS ONE 8: e66318. 23869203 10.1371/journal.pone.0066318 PMC3712023

[68] Newbold RR (2004) Lessons learned from perinatal exposure to diethylstilbestrol. Toxicol Appl Pharmacol 199: 142–150. 15313586 10.1016/j.taap.2003.11.033

[69] Doyle TJ, Bowman JL, Windell VL, McLean DJ, Kim KH (2013) Transgenerational Effects of Di-(2-ethylhexyl) Phthalate on Testicular Germ Cell Associations and Spermatogonial Stem Cells in Mice. Biol Reprod 88: 112. 23536373 10.1095/biolreprod.112.106104 PMC4013901

[70] Stouder C, Paoloni-Giacobino A (2010) Transgenerational effects of the endocrine disruptor vinclozolin on the methylation pattern of imprinted genes in the mouse sperm. Reproduction 139: 373–379. 19887539 10.1530/REP-09-0340

[71] Salian S, Doshi T, Vanage G (2009) Perinatal exposure of rats to Bisphenol A affects the fertility of male offspring. Life Sci 85: 742–752. 19837096 10.1016/j.lfs.2009.10.004

[72] Burdge GC, Hoile SP, Uller T, Thomas NA, Gluckman PD, et al (2011) Progressive, Transgenerational Changes in Offspring Phenotype and Epigenotype following Nutritional Transition. PLoS ONE 6: e28282. 22140567 10.1371/journal.pone.0028282 PMC3227644

[73] Dias BG, Ressler KJ (2014) Parental olfactory experience influences behavior and neural structure in subsequent generations. Nat Neurosci 17: 89–96. 24292232 10.1038/nn.3594 PMC3923835

[74] Greer EL, Maures TJ, Ucar D, Hauswirth AG, Mancini E, et al (2011) Transgenerational epigenetic inheritance of longevity in Caenorhabditis elegans. Nature 479: 365–371. 22012258 10.1038/nature10572 PMC3368121

[75] Ruden DM, Lu X (2008) Hsp90 affecting chromatin remodeling might explain transgenerational epigenetic inheritance in Drosophila. Curr Genomics 9: 500–508. 19506739 10.2174/138920208786241207 PMC2691676

[76] Hauser MT, Aufsatz W, Jonak C, Luschnig C (2011) Transgenerational epigenetic inheritance in plants. Biochim Biophys Acta 1809: 459–468. 21515434 10.1016/j.bbagrm.2011.03.007 PMC4359895

[77] Baker TR, Peterson RE, Heideman W (2014) Using Zebrafish as a Model System for Studying the Transgenerational Effects of Dioxin. Toxicol Sci. 10.1093/toxsci/kfu006 PMC3975160 24470537

[78] Rassoulzadegan M, Grandjean V, Gounon P, Vincent S, Gillot I, et al (2006) RNA-mediated non-mendelian inheritance of an epigenetic change in the mouse. Nature 441: 469–474. 16724059 10.1038/nature04674

[79] Wagner KD, Wagner N, Ghanbarian H, Grandjean V, Gounon P, et al (2008) RNA induction and inheritance of epigenetic cardiac hypertrophy in the mouse. Dev Cell 14: 962–969. 18539123 10.1016/j.devcel.2008.03.009

[80] Pembrey ME (2010) Male-line transgenerational responses in humans. Hum Fertil (Camb) 13: 268–271. 21117937 10.3109/14647273.2010.524721

[81] Meredith S, Dudenhoeffer G, Jackson K (2000) Classification of small type B/C follicles as primordial follicles in mature rats. J Reprod Fertil 119: 43–48. 10864812 10.1530/jrf.0.1190043

[82] Nilsson EE, Schindler R, Savenkova MI, Skinner MK (2011) Inhibitory actions of Anti-Mullerian Hormone (AMH) on ovarian primordial follicle assembly. PLoS ONE 6: e20087. 21637711 10.1371/journal.pone.0020087 PMC3103528

[83] Tateno H, Kimura Y, Yanagimachi R (2000) Sonication per se is not as deleterious to sperm chromosomes as previously inferred. Biol Reprod 63: 341–346. 10859277 10.1095/biolreprod63.1.341

[84] Ward WS, Kimura Y, Yanagimachi R (1999) An intact sperm nuclear matrix may be necessary for the mouse paternal genome to participate in embryonic development. Biol Reprod 60: 702–706. 10026119 10.1095/biolreprod60.3.702

[85] Carvalho B, Irizarry R (2010) A framework for oligonucleotide microarray preprocessing. Bioinformatics: 1367–4803. 10.1093/bioinformatics/btq431 PMC2944196 20688976

