# Supplementary figures and images for: Pesticide Methoxychlor Promotes the Epigenetic Transgenerational Inheritance of Adult-Onset Disease through the Female Germline
Source: PLoS One. 2014 Jul 24;9(7):e102091. doi: 10.1371/journal.pone.0102091 (PMC4109920; doi:10.1371/journal.pone.0102091)

Supplemental Figure S1

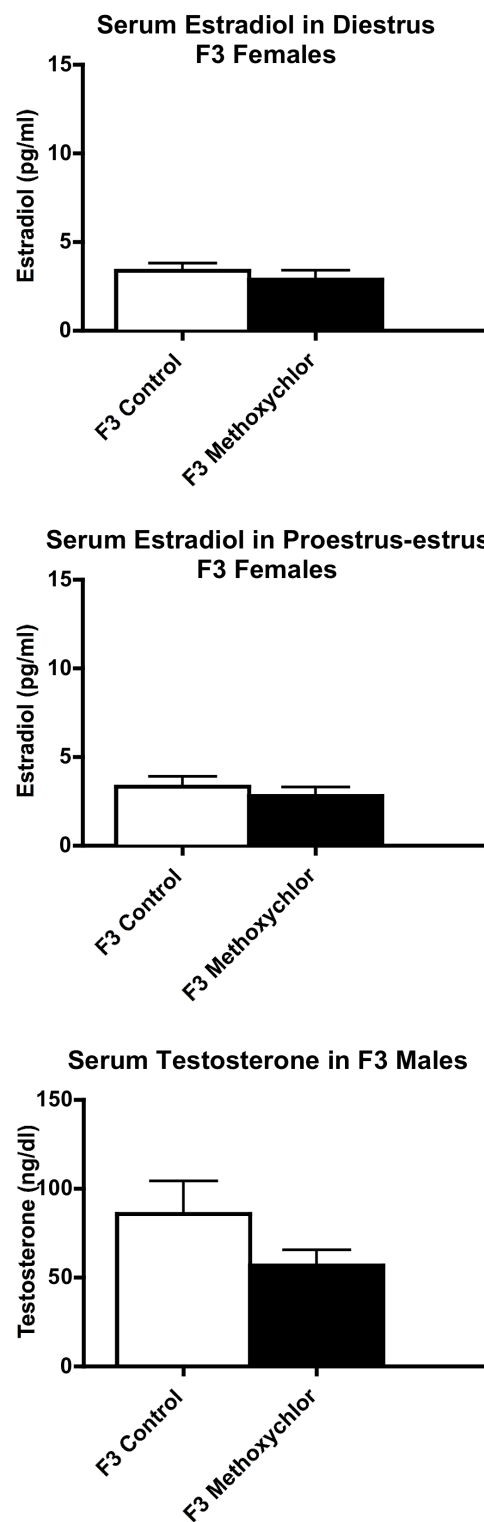

Supplement: Figure S1 — Transgenerational animal hormone levels. A. Serum estradiol concentrations in proestrus-estrus in F3 generation control and methoxychlor lineage females. B. Serum estradiol concentrations in diestrus in F3 generation control and methoxychlor lineage females. C. Serum testosterone concentrations in the F3 generation control and methoxychlor lineage males. (PDF) [file pone.0102091.s001.pdf]

Supplemental Figure S2 (Color)

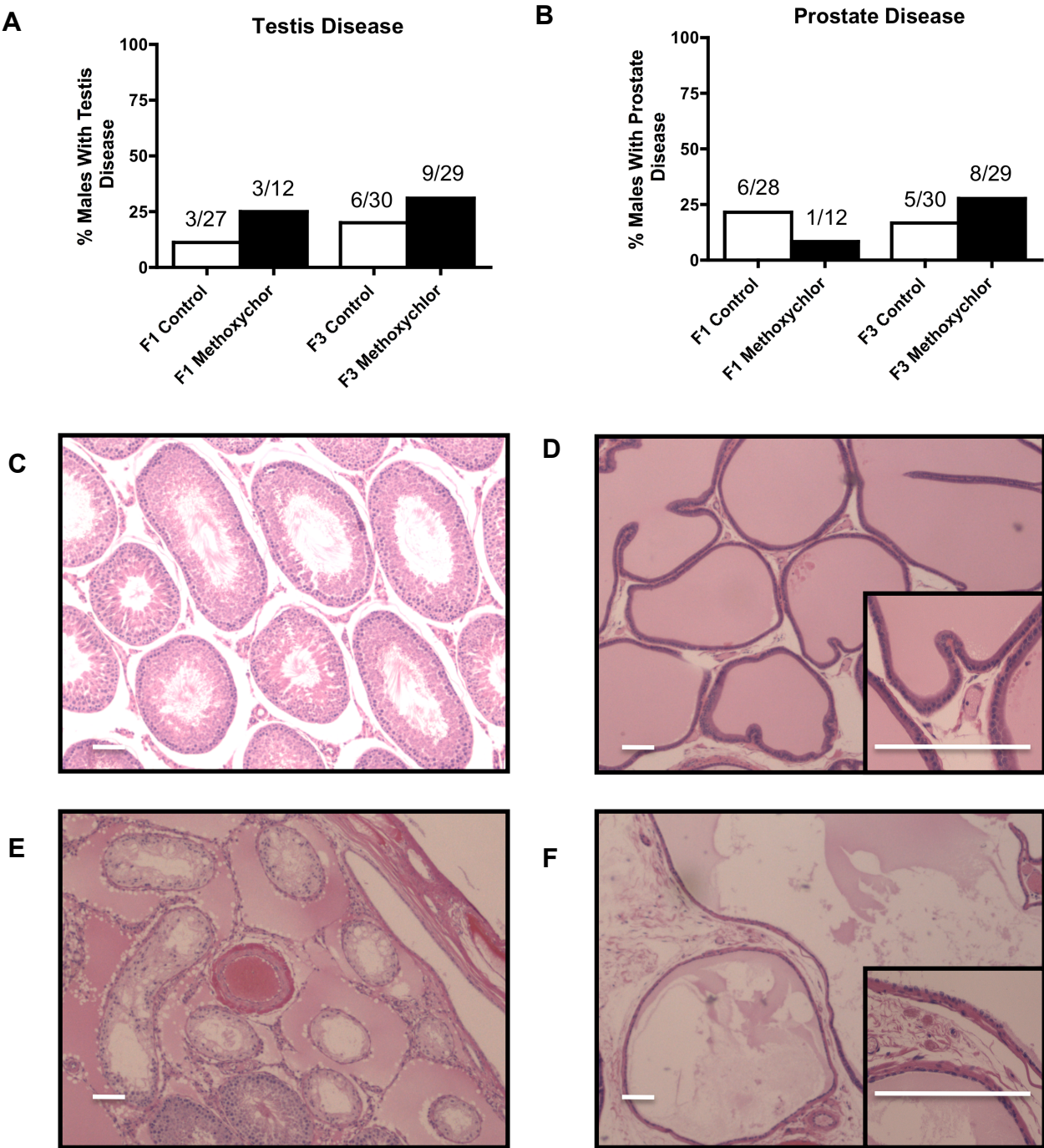

Supplement: Figure S2 — Ancestral exposure to methoxychlor and adult-onset transgenerational testis disease. Percentages of the F1 and F3 generation males of control and methoxychlor lineages with testis disease (panel A) or prostate disease (panel B). The number of diseased rats / total number of rats is shown above the respective bar graphs. Micrographs (scale bar = 100 µm) show testis disease (control: panel C; methoxychlor: panel E) and prostate disease (control: panel D; methoxychlor: panel F) in F3 generation rats. (PDF) [file pone.0102091.s002.pdf]

Supplemental Figure S3

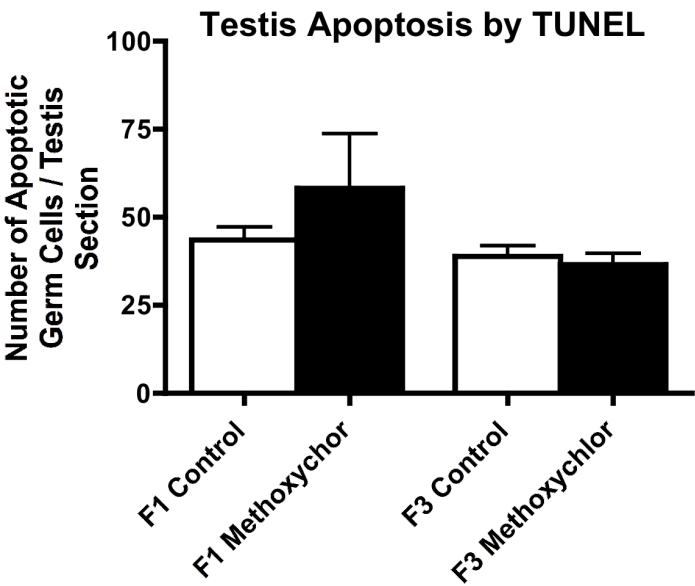

Supplement: Figure S3 — Testicular spermatogenic cell apoptosis. Number of apoptotic germ cells in F1 and F3 generation control (open bars) and methoxychlor (black bars) lineages evaluated by TUNEL assay. (PDF) [file pone.0102091.s003.pdf]
