# Supplementary material for: Pesticide Methoxychlor Promotes the Epigenetic Transgenerational Inheritance of Adult-Onset Disease through the Female Germline
Source: PLoS One. 2014 Jul 24;9(7):e102091. doi: 10.1371/journal.pone.0102091 (PMC4109920; doi:10.1371/journal.pone.0102091)
Supplement: Table S1 — (A) Body Weight and organ weights in F1 and F3 generation female rats of Control and Methoxychlor lineages (mean ± standard error). Asterisks (*, **, ***), if present, indicate statistically significant differences between means of Control and Methoxychlor lineages (P<0.05, P<0.01 and P<0.001 respectively); nd = not determined. (B) Body weight (grams) and organ weights (% of body weight) in F1 and F3 generation male rats of Control and Methoxychlor lineages (mean ± standard error). Asterisks (*, **), if present, indicate statistically significant differences between means of Control and Methoxychlor lineages (P<0.05, P<0.01 respectively); nd = not determined. (PDF) [file pone.0102091.s004.pdf]

Supplemental Table S1

A

Generation	Treatment	Sex	Body Weight (grams)	Ovaries (% BW)	Uterus (% BW)	Kidney (% BW)
F1	Control	F	323.0 ± 6.67	0.0492 ± 0.0012	0.2832 ± 0.0191	0.3289 ± 0.0054
F1	Methoxychlor	F	318.3 ± 6.31	0.0486 ± 0.0021	0.2847 ± 0.0268	0.3523** ± 0.0083
F3	Control	F	289.3 ± 5.01	nd	nd	nd
F3	Methoxychlor	F	288.9 ± 4.32	nd	nd	nd

Body Weight and organ weights in F1 and F3 generation female rats of Control and Methoxychlor lineages (mean ± standard error).Asterisks (*, **, ***), if present, indicate statistically significant differences between means of Control and Methoxychlor lineages ($P<0.05$, $P<0.01$ and $P<0.001$ respectively); nd = not determined.

B

Generation	Treatment	Sex	Body Weight (grams)	Testis (% BW)	Prostate (% BW)	Seminal Vesicle (% BW)	Epididymis (% BW)	Kidney (% BW)
F1	Control	M	567.2 ± 6.83	0.7799 ± 0.0127	0.1943 ± 0.0078	0.0835 ± 0.0020	0.2566 ± 0.0037	0.3415 ± 0.0077
F1	Methoxychlor	M	551.9 ± 8.90	0.7017** ± 0.0193	0.1838 ± 0.0085	0.0812 ± 0.0036	0.2553 ± 0.0058	0.3738* ± 0.0110
F3	Control	M	513.7 ± 8.79	nd	nd	nd	nd	nd
F3	Methoxychlor	M	522.7 ± 7.11	nd	nd	nd	nd	nd

Body weight (grams) and organ weights (% of body weight) in F1 and F3 generation male rats of Control and Methoxychlor lineages (mean ± standard error). Asterisks (*, **), if present, indicate statistically significant differences between means of Control and Methoxychlor lineages ($P<0.05$, $P<0.01$ respectively); nd = not determined.
