# Supplementary material for: Pesticide Methoxychlor Promotes the Epigenetic Transgenerational Inheritance of Adult-Onset Disease through the Female Germline
Source: PLoS One. 2014 Jul 24;9(7):e102091. doi: 10.1371/journal.pone.0102091 (PMC4109920; doi:10.1371/journal.pone.0102091)
Supplement: Table S2 — (A) Individual disease incidence in F1 generation female rats of Control and Methoxychlor lineages. (B) Individual disease incidence in F1 generation male rats of Control and Methoxychlor lineages. (PDF) [file pone.0102091.s005.pdf]

Supplemental Table S2

A. Individual disease incidence in F1 generation female rats of Control and Methoxychlor lineages.

Serial Number	Rat ID	Puberty	Ovary	Uterus	Kidney	Obesity	Tum or	Total Disease
C1	MCTT2-1-1-1	-		-	-	-	-	
C2	MCTT2-1-1-2	-		+	-	-	-	1
C3	MCWW0-1-2-1	-		-		-	+	1
C4	MCWW0-1-2-2	-	-	-	-	-	-	
C5	MCWW0-1-2-3	-	-	-	-	-	-	
C6	MCWW0-1-2-4	-	-	-	-	-	-	
C7	MCAA0-1-3-1	-	-	-	-	-	-	
C8	MCAA0-1-3-2	-		-	-	-	-	
C9	MCAA0-1-3-3	-	-	+	-	-	-	1
C10	MCAA0-1-3-4	-		+	-	-	-	1
C11	MCGG1-1-4-1	-	-	-	-	+	-	1
C12	MCGG1-1-4-2	-	-	-		+	-	1
C13	MCGG1-1-4-3	-	-	-		-	+	1
C14	MCGG1-1-4-4	-	-	-		-	-	
C15	MCWW0-1-6-1	-	-	-	-	-	-	
C16	MCWW0-1-6-2	-		-	-	-	-	
C17	MCWW0-1-6-3	-		-	-	-	-	
C18	MCWW0-1-6-4	-		-	-	-	-	
C19	MCGG2-1-7-1	-		-	-	-	-	
C20	MCGG2-1-7-2	-		-	-	-	-	
C21	MCGG2-1-7-3	-		-	-	-	-	
C22	MCGG2-1-7-4	-		-	+	-	-	1
C23	MCZZ0-1-8-1	-		-	-	-	-	
C24	MCZZ0-1-8-2	-		+	+	-	-	2
C25	MCZZ0-1-8-3	-		-	-	+	-	1
C26	MCZZ0-1-8-4	-		-	-	-	-	
C27	MCZZ0-1-8-5	-		-	-	+	-	1
M1	MMKK0-1-1-1	-		+	-	-	-	1
M2	MMKK0-1-1-2	-	-	+		+	-	2
M3	MMKK0-1-1-3	-		-	-	-	-	
M4	MMKK0-1-1-2	-		-	-	-	-	
M5	MMKK0-1-1-4	-	+	+	+	+	-	4
M6	MMKK0-1-1-5	-	+	-	+	+	-	3
M7	MMGG2-1-2-1	-	-	+	-	-	-	1
M8	MMGG2-1-2-3	-	+	-	-	-	-	1
M9	MMGG2-1-2-4	-	-	+	-	-	-	1
M10	MMGG2-1-2-5	-	-	-	+	-	-	1
M11	MMKK0-1-3-1	-	+	-	-	-	-	1
M12	MMKK0-1-3-2	-	-	-	+	-	-	1
M13	MMKK0-1-3-3	-	-	-	-	-	-	
M14	MMKK0-1-3-4	-		-	-	-	-	
M15	MMKK0-1-3-5	-		-	+	-	-	1
M16	MMJJ0-1-4-1	-		-	+	-	-	1
M17	MMJJ0-1-4-2	-		-	+	-	-	1
M18	MMJJ0-1-4-3	+		-	-	-	-	1
M19	MMJJ0-1-4-4	+		-	-	-	-	1

B. Individual disease incidence in F1 generation male rats of Control and Methoxychlor lineages.

Serial Number	Rat ID	Puberty	Testis	Prostate	Kidney	Obesity	Tumor	Total Disease
C1	MCTT2-1-1-6	-	-	+	-	-	-	1
C2	MCTT2-1-1-7	-	+	-	+	-	-	2
C3	MCTT2-1-1-8	-	+	-	-	-	-	1
C4	MCTT2-1-1-9	-	-	-	-	-	-	
C5	MCWW0-1-2-10	-	-	-	-	-	-	
C6	MCWW0-1-2-11	-		-	-	-	-	
C7	MCAA0-1-3-6	-	-	-	+	-	-	1
C8	MCAA0-1-3-7	-	-	+	-	-	-	1
C9	MCAA0-1-3-8	-	-	-	+	-	-	1
C10	MCAA0-1-3-9	-	-	-	+	-	-	1
C11	MCGG1-1-4-5	-	-	+	-	-	-	1
C12	MCGG1-1-4-6	-	-	-	-	-	-	
C13	MCGG1-1-4-7	-	-	-	+	-	-	1
C14	MCGG1-1-4-8	-	-	-	-	-	-	
C15	MCWW0-1-6-7	-	-	+	-	+	-	2
C16	MCWW0-1-6-8	-	-	-	-	-	-	
C17	MCWW0-1-6-9	-	-	-	-	-	-	
C18	MCWW0-1-6-10	-	-	-	-	-	-	
C19	MCWW0-1-6-11	-			-	-	-	
C20	MCWW0-1-6-12	-	-	+	-	-	-	1
C21	MCGG2-1-7-6	-	-	-	-	-	-	
C22	MCGG2-1-7-7	-	-	-	-	-	-	
C23	MCGG2-1-7-8	-	-	-	-	-	-	
C24	MCGG2-1-7-9	-	-	-	-	-	-	
C25	MCZZ0-1-8-6	-	-	-	-	-	-	
C26	MCZZ0-1-8-7	-	-	-	-	-	-	
C27	MCZZ0-1-8-8	-	-	-	-	+	-	1
C28	MCZZ0-1-8-9	-	+	-	-	+	-	2
C29	MCZZ0-1-8-10	+	-	+	-	-	-	2
M1	MMKK0-1-1-6	-	-	-	+	-	-	1
M2	MMKK0-1-1-7	-	+	-	-	-	-	1
M3	MMKK0-1-1-8	-	+	-	-	-	-	1
M4	MMGG2-1-2-6	-	-	-	+	-	-	1
M5	MMGG2-1-2-7	-	-	-	+	-	-	1
M6	MMGG2-1-2-8	+	-	+	-	-	-	2
M7	MMKK0-1-3-6	-	-	-	-	-	-	
M8	MMKK0-1-3-7	-	-	-	-	-	-	
M9	MMKK0-1-3-8	-	-	-	+	-	-	1
M10	MMJJ0-1-4-6	-	-	-	+	-	-	1
M11	MMJJ0-1-4-7	-	-	-	+	-	-	1
M12	MMJJ0-1-4-8	-	+	-	+	-	-	2
