# Supplementary material for: Pesticide Methoxychlor Promotes the Epigenetic Transgenerational Inheritance of Adult-Onset Disease through the Female Germline
Source: PLoS One. 2014 Jul 24;9(7):e102091. doi: 10.1371/journal.pone.0102091 (PMC4109920; doi:10.1371/journal.pone.0102091)
Supplement: Table S3 — (A) Individual disease incidence in F3 generation female rats of Control and Methoxychlor lineages. (B) Individual disease incidence in F3 generation male rats of Control and Methoxychlor lineages. (PDF) [file pone.0102091.s006.pdf]

Supplemental Table S3

A. Individual disease incidence in F3 generation female rats of Control and Methoxychlor lineages.

Serial #	Rat ID	Puberty	Ovary	Uterus	Kidney	Tumor	Obesity	Total Disease
C1	MCTT2-3-1-1	-	-	-	+	-	-	1
C2	MCTT2-3-1-2	-	-	+	-	-	-	1
C3	MCGG1-3-2-1	+	-	-	-	-	-	1
C4	MCGG1-3-2-2	+	-	+	-	-	-	2
C5	MCGG1-3-3-1	-	-	-	-	-	-	
C6	MCGG1-3-3-2	-	-	-	-	-	-	
C7	MCWW0-3-4-1	-	-	-	-	-	-	
C8	MCWW0-3-4-2	-	-	-	-	-	-	
C9	MCGG1-3-6-1	-	-	-	-	-	-	
C10	MCAA0-3-7-1	-	-	-	-	-	-	
C11	MCAA0-3-7-2	-		-	-	-	+	1
C12	MCGG2-3-8-1	-	-	-	+	-	-	1
C13	MCGG2-3-8-2	-		-	+	-	-	1
C14	MCTT2-3-9-1	-	-	-	+	-	-	1
C15	MCTT2-3-9-2	-		+	+	-	-	2
C16	MCWW0-3-10-1	-	-	-	-	-	-	
C17	MCGG2-3-12-1	-		-	-	-	-	
C18	MCGG1-3-13-1	-		-	-	-	-	
C19	MCGG1-3-14-1	-		+	-	-	-	1
C20	MCGG1-3-15-1	-		-	-	-	-	
C21	MCWW0-3-16-1	-	-	-	-	-	-	
C22	MCWW0-3-16-2	-		-	-	-	-	
C23	MCGG1-3-17-1	-	-	-	-	-	-	
C24	MCAA0-3-18-1	-	-	-	-	-	-	
C25	MCAA0-3-19-1	-	-	+	-	-	-	1
C26	MCAA0-3-19-2	-	-	+	-	-	-	1
C27	MCGG2-3-20-1	-	-	-	-	-	-	
C28	MCAA0-3-21-1	-	-	-	-	-	-	
C29	MCGG1-3-22-1	-		-	-	-	-	
M1	MMKK0-3-1-1	-	+	-	+	-	-	2
M2	MMKK0-3-1-2	-		-	-	-	-	
M3	MMKK0-3-2-1	-	+	-	-	-	-	1
M4	MMKK0-3-2-2	-	+	-	-	-	-	1
M5	MMGG2-3-3-1	-	+	-	+	-	-	2
M6	MMKK0-3-4-1	-	+	-	+	-	+	3
M7	MMKK0-3-5-1	-	-	-	-	-	-	
M8	MMKK0-3-5-2	-	-	+	-	-	-	1
M9	MMGG2-3-6-1	-		-	-	-	-	
M10	MMGG2-3-6-2	-	-	-	-	-	-	
M11	MMKK0-3-7-1	-	+	-	+	-	+	3
M12	MMKK0-3-7-2	-	-	-	+	-	+	2
M13	MMKK0-3-8-1	-	+	-	-	-	-	1
M14	MMJJ0-3-9-1	-		-	+	-	-	1
M15	MMJJ0-3-9-2	-	-	-	+	-	-	1
M16	MMKK0-3-10-1	-	-	-	-	-	-	

M17	MMKK0-3-10-2	-	+	-	-	-	-	1
M18	MMKK0-3-11-1	-	-	-	+	-	-	1
M19	MMKK0-3-12-1	-	-	-	+	-	-	1
M20	MMKK0-3-13-1	-	+	+	+	-	-	3
M21	MMKK0-3-13-2	-	+	+	+	-	-	3
M22	MMKK0-3-13-3	-	+	-	+	-	-	2
M23	MMJJ0-3-14-1	+	+	-	+	-	-	3
M24	MMJJ0-3-14-2	+		-	-	-	-	1
M25	MMKK0-3-15-1	-		-	+	-	+	2
M26	MMKK0-3-15-2	-	+	-	-	-	+	2
M27	MMKK0-3-15-3	-		-	+	-	+	2
M28	MMJJ0-3-16-1	-		-	+	-	+	2
M29	MMJJ0-3-16-2	-		-	+	-	-	1
M30	MMJJ0-3-16-3	-		-	-	-	+	1

B. Individual disease incidence in F3 generation male rats of Control and Methoxychlor lineages.

Serial #	Rat ID	Puberty	Testis	Prostate	Kidney	Tumor	Obesity	Total Disease
C1	MCTT2-3-1-5	-	-	-	-	-	-	
C2	MCTT2-3-1-6	-	-	-	-	-	-	
C3	MCGG1-3-2-10	-	-	-	-	-	-	
C4	MCGG1-3-2-11	-	-	-	-	-	-	
C5	MCGG1-3-3-6	-	+	-	-	-	-	1
C6	MCGG1-3-3-7	-	+	-	-	-	-	1
C7	MCWW0-3-4-4	-	-	+	+	-	+	3
C8	MCWW0-3-4-5	-	-	-	-	-	-	
C9	MCGG1-3-6-8	-	-	+	+	-	-	2
C10	MCAA0-3-7-6	-	-	-	-	-	-	
C11	MCAA0-3-7-7	-	-	-	-	-	-	
C12	MCGG2-3-8-7	-	+	-	-	-	-	1
C13	MCGG2-3-8-8	-	-	-	-	-	-	
C14	MCTT2-3-9-5	-	-	-	-	-	+	1
C15	MCTT2-3-9-6	-	-	-	-	-	+	1
C16	MCWW0-3-10-9	-	+	-	-	-	-	1
C17	MCWW0-3-10-10	-	-	-	-	-	+	1
C18	MCGG2-3-12-4	-	-	-	-	-	+	1
C19	MCGG1-3-13-5	-	-	+	-	-	-	1
C20	MCGG1-3-14-4	-	-	-	-	-	-	
C21	MCGG1-3-15-3	-	+	-	-	-	-	1
C22	MCWW0-3-16-5	-	-	+	-	-	-	1
C23	MCWW0-3-16-6	-	-	-	-	-	-	
C24	MCGG1-3-17-4	+	-	-	-	-	-	1
C25	MCAA0-18-2	-	-	-	-	-	-	
C26	MCAA0-3-19-5	-	-	-	-	-	+	1
C27	MCAA0-3-19-6	-	+	-	+	-	-	2
C28	MCGG2-3-20-9	-	-	-	-	-	+	1
C29	MCGG2-3-20-10	-				+	-	1
C30	MCAA0-3-21-5	-	-	+	-	-	-	1
C31	MCGG1-3-22-8	-	-	-	-	-	-	
M1	MMKK0-3-1-5	-	-	-	-	-	+	1
M2	MMKK0-3-1-6	-	+	-	-	-	-	1
M3	MMKK0-3-2-7	-	+	+	-	-	-	2
M4	MMKK0-3-2-8	-	-	-	-	-	-	
M5	MMGG2-3-3-7	-	+	-	-	-	-	1
M6	MMKK0-3-4-8	-	-	-	-	-	-	
M7	MMKK0-3-5-6	-	-	-	-	-	-	
M8	MMKK0-3-5-7	-	+	+	-	-	-	2
M9	MMGG2-3-6-6	-	-	-	-	-	+	1
M10	MMGG2-3-6-7	-	-	-	+	-	+	2
M11	MMKK0-3-7-6	-	-	-	+	-	+	2
M12	MMKK0-3-7-7	-	-	+	-	-	+	2
M13	MMKK0-3-8-2	-	+	-	+	-	-	2
M14	MMJJ0-3-9-4	-	-	+	-	-	+	2
M15	MMJJ0-3-9-5	-	-	+	+	-	+	3
M16	MMKK0-3-10-7	-	-	+	+	-	+	3

M17	MMKK0-3-10-8	-	-	+	-	-	+	2
M18	MMKK0-3-11-10	-	+	-	+	-	+	3
M19	MMKK0-3-12-5	-	+	-	+	-	+	3
M20	MMKK0-3-13-10	-	-	-	-	-	-	
M21	MMKK0-3-13-11	-	-	-	-	-	-	
M22	MMKK0-3-13-9	-	+	-	-	-	-	1
M23	MMJJ0-3-14-7	+	+	+	-	-	-	3
M24	MMKK0-3-15-7	-	-	-	-	-	-	
M25	MMKK0-3-15-8	-	-	-	-	-	-	
M26	MMKK0-3-15-9	-	-	-	-	-	-	
M27	MMJJ0-3-16-10	-	-	-	-	-	+	1
M28	MMJJ0-3-16-11	-	-	-	-	-	-	
M29	MMJJ0-3-16-12	-	-	-	+	-	+	2
