# Supplementary material for: Pesticide Methoxychlor Promotes the Epigenetic Transgenerational Inheritance of Adult-Onset Disease through the Female Germline
Source: PLoS One. 2014 Jul 24;9(7):e102091. doi: 10.1371/journal.pone.0102091 (PMC4109920; doi:10.1371/journal.pone.0102091)
Supplement: Table S4 — (A) Body Weight in F4 generation Outcross and Reverse Outcross female rats of Control and Methoxychlor lineages (mean ± standard error). (B) Body weight (grams) in F4 generation Outcross and Reverse Outcross male rats of Control and Methoxychlor lineages (mean ± standard error). (PDF) [file pone.0102091.s007.pdf]

Supplemental Table S4

A. Body Weight in F4 generation Outcross and Reverse Outcross female rats of Control and Methoxychlor lineages (mean ± standard error).

Generation	Lineage	Sex	Body Weight (grams)
F4	Outcross Control	F	291.7 ± 3.25
F4	Outcross Methoxychlor	F	297.8 ± 4.86
F4	Reverse Outcross Control	F	294.9 ± 4.89
F4	Reverse Outcross Methoxychlor	F	294.4 ± 3.15

B. Body weight (grams) in F4 generation Outcross and Reverse Outcross male rats of Control and Methoxychlor lineages (mean ± standard error).

Generation	Lineage	Sex	Body Weight (grams)
F4	Outcross Control	M	533.8 ± 7.44
F4	Outcross Methoxychlor	M	519.5 ± 9.93
F4	Reverse Outcross Control	M	507.7 ± 5.53
F4	Reverse Outcross Methoxychlor	M	519.9 ± 6.58
