# Supplementary material for: Pesticide Methoxychlor Promotes the Epigenetic Transgenerational Inheritance of Adult-Onset Disease through the Female Germline
Source: PLoS One. 2014 Jul 24;9(7):e102091. doi: 10.1371/journal.pone.0102091 (PMC4109920; doi:10.1371/journal.pone.0102091)
Supplement: Table S5 — (A) Individual disease incidence in F4 generation Outcross female rats of Control and Methoxychlor lineages. (B) Individual disease incidence in F4 generation Outcross male rats of Control and Methoxychlor lineages. (PDF) [file pone.0102091.s008.pdf]

Supplemental Table S5

A. Individual disease incidence in F4 generation Outcross female rats of Control and Methoxychlor lineages.

Serial Number	Rat ID	Puberty	Ovary	Uterus	Kidney	Tumor	Obesity	Total Disease
C1	MOC5-O4-1-1	-	-	-	-	-	-	
C2	MOC5-O4-1-2	-	-	-	-	-	-	
C3	MOC5-O4-1-3	-	-	-	-	-	-	
C4	MOC5-O4-1-4	-	-	-	+	-	-	1
C5	MOC5-O4-1-5	+	-	-	+	-	-	2
C6	MOC5-O4-1-6	+	-	-	-	-	-	1
C7	MOC5-O4-1-7	-	-	-	+	-	-	1
C8	MOC4-O4-2-1	-	-	-	-	-	-	
C9	MOC4-O4-2-2	-	-	-	-	-	+	1
C10	MOC4-O4-2-3	-	-	-	-	-	-	
C11	MOC4-O4-2-4	-	+	-	+	-	-	2
C12	MOC4-O4-2-6	-		-		-	+	1
C13	MOC4-O4-2-7	-		-		-	+	1
C14	MOC7-04-3-1	-		-		+	-	1
C15	MOC7-04-3-3	-		+		-	-	1
C16	MOC8-O4-4-1	-	-	-	-	-	-	
C17	MOC8-O4-4-2	-	-	-	-	-	-	
C18	MOC2-O4-5-1	-	+	-	-	-	-	1
C19	MOC2-O4-5-2	-		-		-	+	1
C20	MOC2-O4-5-3	-		-		-	+	1
C21	MOC2-O4-5-6	-		-		-	+	1
C22	MOC1-04-6-1	-		+		-	-	1
C23	MOC1-04-6-2	-		+		-	-	1
C24	MOC6-O4-7-1	-	-	-	-	-	+	1
C25	MOC6-O4-7-3	-		-		-	+	1
M1	MOM7-04-1-2	-		+	+	+	-	3
M2	MOM7-04-1-3	-		-	+	-	-	1
M3	MOM5-04-2-1	-		-		-	+	1
M4	MOM5-04-2-3	-		-		-	+	1
M5	MOM3-O4-3-2	-	-	+	-	-	-	1
M6	MOM3-O4-3-3	-	+	-	-	-	-	1
M7	MOM3-O4-3-4	-	+	-	-	-	-	1
M8	MOM3-O4-3-5	-	-	-	-	-	+	1
M9	MOM8-O4-5-1	-	-	-	-	-	-	
M10	MOM8-O4-5-2	-	-	-	-	-	+	1
M11	MOM8-O4-5-3	-	+	-	-	-	+	2
M12	MOM8-O4-5-4	-	-	-	+	-	-	1
M13	MOM6-O4-7-1	-	-	-	-	-	-	
M14	MOM6-O4-7-2	-	+	-	-	-	-	1
M15	MOM6-O4-7-4	-	-	-	-	-	-	
M16	MOM6-O4-7-5	-	-	-	+	-	+	2
M17	MOM6-O4-7-6	-	-	-	+	-	+	2
M18	MOM6-O4-7-7	-	-	-	+	-	+	2
M19	MOM2-O4-8-1	-	-	-	-	-	+	1

B. Individual disease incidence in F4 generation Outcross male rats of Control and Methoxychlor lineages.

Serial Number	Rat ID	Puberty	Testis	Kidney	Tumor	Obesity	Total Disease
C1	MOC5-O4-1-9	-	-	-	-	-	
C2	MOC5-O4-1-10	-	+	+	-	-	2
C3	MOC5-O4-1-11	-	-	-	-	+	1
C4	MOC5-O4-1-12	-	-	+	-	-	1
C5	MOC4-O4-2-9	-	-	-	-	-	
C6	MOC4-O4-2-10	-	-	-	-	-	
C7	MOC4-O4-2-11	-	-	-	-	-	
C8	MOC4-O4-2-12	-	-	-	-	-	
C9	MOC4-O4-2-13	-	+	-	-	-	1
C10	MOC7-O4-3-6	-	-	+	-	+	2
C11	MOC7-O4-3-7	-		+	-	-	1
C12	MOC7-O4-3-8	-			-	+	1
C13	MOC7-O4-3-9	-		+	-	-	1
C14	MOC7-O4-3-11	-			-	+	1
C15	MOC8-04-4-5	-			-	+	1
C16	MOC8-04-4-6	-			-	+	1
C17	MOC2-04-5-10	-		+	-	-	1
C18	MOC2-04-5-11	-		+	-	-	1
C19	MOC1-04-6-5	-		+	-	+	2
C20	MOC1-04-6-6	-		+	-	-	1
C21	MOC1-04-6-7	-		+	-	-	1
C22	MOC1-04-6-8	-			-	+	1
C23	MOC1-04-6-9	-			-	+	1
C24	MOC1-04-6-10	-			-	+	1
C25	MOC6-O4-7-6	-	-	+	-	-	1
C26	MOC6-O4-7-7	-	-	-	-	+	1
C27	MOC6-O4-7-8	-	-	-	-	+	1
C28	MOC6-O4-7-9	-	-	+	-	-	1
C29	MOC6-O4-7-10	-	-	-	-	-	
M1	MOM7-O4-1-8	-	+	-	-	-	1
M2	MOM7-O4-1-9	-	-	-	-	-	
M3	MOM5-O4-2-7	-	+	-	-	-	1
M4	MOM5-O4-2-8	-	+	+	-	-	2
M5	MOM5-O4-2-9	-	-	-	-	-	
M6	MOM3-O4-3-10	-	-	-	-	-	
M7	MOM3-O4-3-11	-	-	+	-	-	1
M8	MOM3-O4-3-12	-	-	-	-	+	1
M9	MOM1-04-4-9	-			-	+	1
M10	MOM1-04-4-10	-			-	+	1
M11	MOM1-04-4-11	-			-	+	1
M12	MOM8-O4-5-8	-	-	+	-	-	1
M13	MOM8-O4-5-9	-	-	+	-	-	1
M14	MOM8-O4-5-10	-	-	-	-	-	
M15	MOM8-O4-5-11	-			-	+	1
M16	MOM8-O4-5-12	-	-	+	-	+	2
M17	MOM4-O4-6-9	-	-	+	-	-	1
M18	MOM4-O4-6-10	-	-	-	-	+	1
M19	MOM4-O4-6-11	-	-	+	-	+	2
M20	MOM6-04-7-12	+	-	+	-	-	2
M21	MOM2-04-8-2	-			-	+	1
M22	MOM2-04-8-3	-			-	+	1
