# Supplementary material for: Pesticide Methoxychlor Promotes the Epigenetic Transgenerational Inheritance of Adult-Onset Disease through the Female Germline
Source: PLoS One. 2014 Jul 24;9(7):e102091. doi: 10.1371/journal.pone.0102091 (PMC4109920; doi:10.1371/journal.pone.0102091)
Supplement: Table S6 — (A) Individual disease incidence in F4 generation Reverse Outcross female rats of Control and Methoxychlor lineages. (B) Individual disease incidence in F4 generation Reverse Outcross male rats of Control and Methoxychlor lineages. (PDF) [file pone.0102091.s009.pdf]

Supplemental Table S6

A. Individual disease incidence in F4 generation Reverse Outcross female rats of Control and Methoxychlor lineages.

Serial Number	Rat ID	Puberty	Ovary	Uterus	Kidney	Tumor	Obesity	Total Disease
C1	MCCGG1-R4-1-1	-	-	-	-	-	-	
C2	MCCGG1-R4-1-2	-	-	-	-	-	-	
C3	MCCGG1-R4-1-3	-	-	+	-	-	-	1
C4	MCTT2-R4-2-1	-	-	+	-	-	+	2
C5	MCTT2-R4-2-2	-	-	+	-	-	+	2
C6	MCTT2-R4-2-4	-		+		-	-	1
C7	MCTT2-R4-2-5	-		+		-	-	1
C8	MCWW0-R4-3-1	-	+	-		-	+	2
C9	MCWW0-R4-3-2	-		+		-	+	2
C10	MCGG1-R4-4-1	+	-	-	-	-	-	1
C11	MCGG1-R4-4-2	+	-	-	-	-	-	1
C12	MCGG1-R4-4-3	-	-	+	-	-	-	1
C13	MCAA0-R4-5-1	-	-	-	-	-	-	
C14	MCAA0-R4-5-2	-	-	-	-	-	-	
C15	MCGG1-R4-6-1	-	-	+	+	-	-	2
C16	MCGG1-R4-6-2	-	-	-	-	-	-	
C17	MCGG1-R4-6-3	-	-	-	-	-	-	
C18	MCGG2-R4-7-1	-	-	-	+	-	-	1
C19	MCGG2-R4-7-2	-	-	-	+	-	-	1
M1	MMGG2-R4-1-1	-	-	+	+	-	-	2
M2	MMGG2-R4-1-2	-	+	-	-	-	-	1
M3	MMKK0-R4-2-1	+	-	+	+	-	-	3
M4	MMKK0-R4-3-2	-	-	+	+	-	-	2
M5	MMKK0-R4-3-3	-	+	-	-	-	-	1
M6	MMKK0-R4-4-1	-		-		+	-	1
M7	MMKK0-R4-4-4	-		+		-	-	1
M8	MMKK0-R4-7-3	-	-	-	+	-	-	1
M9	MMKK0-R4-7-4	-	-	+	+	-	-	2
M10	MMKK0-R4-7-5	-	-	+	+	-	-	2
M11	MMKK0-R4-7-6	-	-	+	+	-	-	2
M12	MMKK0-R4-7-7	-	-	-	+	-	-	1
M13	MMKK0-R4-8-1	-	-	-	-	-	+	1
M14	MMKK0-R4-8-2	-	-	-	+	-	+	2
M15	MMKK0-R4-8-3	-	-	-	+	-	+	2
M16	MMKK0-R4-8-4	-	-	-	+	-	+	2
M17	MMKK0-R4-8-5	-	-	-	-	-	+	1

B. Individual disease incidence in F4 generation Reverse Outcross male rats of Control and Methoxychlor lineages.

Serial Number	Rat ID	Puberty	Testis	Kidney	Tumor	Obesity	Total Disease
C1	MCTT2-R4-2-9	-		+	-	-	1
C2	MCWW0-R4-3-4	-	-	-	-	-	
C3	MCWW0-R4-3-5	-	-	-	-	-	
C4	MCWW0-R4-3-6	-	-	+	-	-	1
C5	MCWW0-R4-3-7	-	-	-	-	-	
C6	MCWW0-R4-3-8	-	+	-	-	-	1
C7	MCWW0-R4-3-9	-	-	-	-	-	
C8	MCGG1-R4-4-6	-	-	-	-	-	
C9	MCGG1-R4-4-7	-	-		-	-	
C10	MCGG1-R4-4-8	-	-	-	-	-	
C11	MCGG1-R4-4-10	-	-	-	-	-	
C12	MCGG1-R4-4-11	-	-	-	-	-	
C13	MCGG1-R4-4-12	-	-	+	-	-	1
C14	MCGG1-R4-6-8	-		+	-	-	1
C15	MCGG1-R4-6-9	-			-	+	1
C16	MCGG2-R4-7-4	-		+	-	-	1
C17	MCGG2-R4-7-6	-	-	+	-	-	1
C18	MCGG2-R4-7-7	-	-	+	-	-	1
C19	MCGG2-R4-7-8	-	-	+	-	-	1
M1	MMGG2-R4-1-6	-	-	+	-	-	1
M2	MMGG2-R4-1-7	-	-	+	-	-	1
M3	MMGG2-R4-1-8	-	-	+	-	-	1
M4	MMGG2-R4-1-9	-	-	+	-	-	1
M5	MMKK0-R4-3-8	-	-	+	-	-	1
M6	MMKK0-R4-3-9	-	+	+	-	-	2
M7	MMKK0-R4-4-9	+	-	+	-	+	3
M8	MMKK0-R4-4-10	-	+	+	-	+	3
M9	MMKK0-R4-4-11	-	-	+	-	+	2
M10	MMGG2-R4-5-6	-			-	+	1
M11	MMGG2-R4-5-8	-			-	+	1
M12	MMKK0-R4-6-7	-			-	+	1
M13	MMKK0-R4-7-9	-	-	+	-	-	1
M14	MMKK0-R4-7-10	-	-	+	-	-	1
M15	MMKK0-R4-8-7	-	-	+	-	+	2
M16	MMKK0-R4-8-8	-	-	+	-	+	2
M17	MMKK0-R4-8-9	-	+	+	-	-	2
M18	MMKK0-R4-8-10	-	+	+	-	+	3
M19	MMKK0-R4-8-13	-			-	+	1
