# Supplementary material for: Pesticide Methoxychlor Promotes the Epigenetic Transgenerational Inheritance of Adult-Onset Disease through the Female Germline
Source: PLoS One. 2014 Jul 24;9(7):e102091. doi: 10.1371/journal.pone.0102091 (PMC4109920; doi:10.1371/journal.pone.0102091)
Supplement: Table S7 — Methoxychlor lineage F3 generation sperm average epimutations. (PDF) [file pone.0102091.s010.pdf]

Supplemental Table S7

Methoxychlor Lineage F3 Generation Sperm Average Epimutations

Chromosome	cSTART	cSTOP	TSS	NCBI gene ID	Gene Name
chr1	108954210	108954810	108955130	29707	Gabra5
chr1	131541261	131541861	131540948	293023	Klhl25
chr1	143415679	143416279	143415815	85431	Nox4
chr1	157945198	157945883	157945870	308857	Dnajb13
chr1	185645938	185646538	185647581	361649	Atxn2l
chr1	203243680	203244280	203244480	24209	Ascl2
chr1	205168543	205169143	205166049	309135	Ano1
chr1	206566558	206567158	206569503	293653	Acy3
chr1	207994578	207995178	207993021	171096	Cst6
chr1	208694707	208695307	208694860	309176	Mrpl49
chr1	209888122	209888817	209888462	171335	Cox8a
chr1	211625999	211626599	211629024	499314	Tut1
chr1	21997763	21998363	21998005	294123	Taar5
chr1	222227402	222228002	222229865	259275	Ostf1
chr1	222271150	222271750	222274597	293871	RGD1311863
chr1	232762359	232763038	232766488	309301	Rcl1
chr1	233363254	233363854	233361107	373544	Ermp1
chr1	246252356	246252956	246252295	361757	Opalin
chr1	248820010	248820610	248816413	309374	Ankrd2
chr1	251275707	251276307	251278221	294007	Pprc1
chr1	253225429	253226029	253228546	114846	Gsto1
chr1	41391892	41392492	41392522	308097	Tagap
chr1	65108322	65108922	65107536	308318	RGD1308782
chr1	72887651	72888741	72885666	361509	Ube2m
chr1	78511795	78512395	78508576	292687	Qpctl
chr1	79833293	79833893	79832891	308429	RGD1565787
chr1	79917004	79917604	79917951	60378	Lypd3
chr1	81266911	81267511	81266827	24300	Cyp2b1
chr1	81778915	81779515	81780087	29295	Cyp2b12
chr1	87132142	87132742	87130690	499129	Kctd15
chr1	94939682	94940282	94942787	292875	Aspdh
chr2	113203764	113204364	113204303	246775	Tnfsf10
chr2	122384576	122385176	122384815	310324	Dcun1d1
chr2	150825988	150826588	150826482	170923	Rap2b
chr2	153733257	153733857	153733281	499630	RGD1565059
chr2	181442601	181443201	181439450	502017	Dpm3
chr2	182790373	182790973	182787370	295214	S100a1
chr2	190419465	190420065	190422646	50654	Ctss
chr2	190652731	190653415	190653151	689432	Mrps21
chr2	198804991	198805591	198807339	60565	Syt6
chr2	199014676	199015276	199014710	365895	Hipk1
chr2	203455748	203456348	203456121	64352	Gstm5
chr2	21046299	21046992	21046303	124323	Rps23
chr2	212465252	212465852	212464764	64443	Gpr88
chr2	226734741	226735732	226732069	64017	Enpep
chr2	235906884	235907582	235907339	310903	Adh6

chr2	239769483	239770083	239767802	310914	Bmpr1b
chr2	258014283	258014883	258014436	362065	Gpr177
chr2	44703563	44704163	44703931	294747	RGD1561161
chr2	45078508	45079108	45079329	78951	Hspb3
chr2	53900491	53901091	53901011	310362	RGD1305938
chr2	88311847	88312524	88309336	499566	Car13
chr2	93381111	93381815	93382152	361916	Chmp4c
chr2	9803782	9804382	9804234	365627	Mblac2
chr3	10579727	10580327	10580780	25698	Ass1
chr3	109741246	109741846	109743344	691966	Sqrdl
chr3	114181958	114182558	114182384	364738	Gabpb1l
chr3	118884297	118884897	118881211	311429	RGD1311267
chr3	143178668	143179268	143178674	24888	Bcl2l1
chr3	153171837	153172437	153172076	362263	Ptprt
chr3	154746395	154746995	154748976	29576	Wisp2
chr3	156275667	156276267	156275540	29182	Cdh22
chr3	157964936	157965536	157967941	296380	Arfgef2
chr3	159773002	159773602	159773643	311658	Nfatc2
chr3	169311014	169311614	169311204	81775	Rps21
chr3	169566659	169567359	169567487	366274	Ntsr1
chr3	170066055	170066655	170064582	296469	Nkain4
chr3	40429016	40429616	40430149	366057	Gapdh-ps1
chr3	4558121	4558721	4557651	306322	Sdccag3
chr3	55817065	55817665	55818634	362149	Cir1
chr3	69364020	69364719	69364756	404888	Olr483
chr3	72453353	72453953	72454364	404841	Olr636
chr3	73453260	73453860	73451888	404824	Olr681
chr3	73938062	73938662	73939193	366112	Olr705
chr3	7456924	7457524	7456260	362094	Mrps2
chr3	75209387	75209987	75210001	295922	Mtch2
chr3	76854377	76855072	76854414	311204	Slc35c1
chr3	8059300	8059900	8059535	499765	RGD1564114
chr3	89819495	89820095	89816787	83617	Hipk3
chr4	104812523	104813201	104812176	282834	Vps24
chr4	134542013	134542613	134541533	65275	Gpr27
chr4	149256959	149257559	149259252	362414	Tada3l
chr4	152757456	152758056	152757539	405214	Olr825
chr4	158955841	158956441	158959285	362429	Mfap5
chr4	160708747	160709347	160712581	312705	C1r
chr4	164676391	164676991	164674386	408222	Senp18
chr4	165760727	165761450	165760257	500331	LOC500331
chr4	177160113	177160713	177162109	497197	Plcz1
chr4	184636443	184637043	184636279	500366	Rep15
chr4	54901508	54902108	54902331	60590	Grm8
chr4	57448670	57449270	57452110	296959	Cpa2
chr4	65997649	65998249	65999405	312248	Ubn2
chr4	76617978	76618578	76621827	436582	Atp6v0e2
chr5	122063213	122063813	122062871	29223	Ak3l1
chr5	135901429	135902029	135901623	170544	Cyp4a1
chr5	136836197	136836797	136834619	619582	Tmem69
chr5	148718180	148718780	148718296	313050	Lck

chr5	153707676	153708276	153707653	170933	Syf2
chr5	160621088	160621688	160621531	313668	Ddi2
chr5	160643337	160644052	160646313	298607	Agmat
chr5	160700556	160701156	160700757	362652	Dnajc16
chr5	163764037	163764637	163762227	25069	Tnfrsf8
chr5	169412678	169413278	169409305	26759	Acot7
chr5	17120943	17121543	17120959	122772	Rps20
chr5	64096338	64096938	64096576	313231	Alg2
chr5	66330524	66331204	66329047	362518	RGD1307218
chr5	66371922	66372620	66371469	313216	Rnf20
chr6	101723802	101724402	101722655	362756	Pigh
chr6	104749340	104749940	104749464	24777	Slc10a1
chr6	108075603	108076203	108075507	681337	Acot4
chr6	108089268	108089868	108089348	314304	Acot3
chr6	115024215	115024815	115024998	25360	Tshr
chr6	126696183	126696982	126694869	63865	Lgmn
chr6	24061917	24062517	24060920	192209	Spdya
chr6	44114675	44115275	44113910	65190	Rsad2
chr6	48264069	48264669	48263991	298936	Sntg2
chr6	54239524	54240124	54240268	25690	Ahr
chr6	75647016	75647616	75649846	29673	Psma6
chr6	95477587	95478187	95475389	114634	Six1
chr6	99555294	99555894	99553851	60661	Max
chr7	10061255	10061951	10060456	117107	Zbtb7a
chr7	10130545	10131145	10129162	64517	Thop1
chr7	10318546	10319146	10319958	252928	Timm13
chr7	10920788	10921388	10920010	299611	Apc2
chr7	10922579	10923179	10920010	299611	Apc2
chr7	10922779	10923469	10920010	299611	Apc2
chr7	113020690	113021290	113018824	353498	Cyp11b3
chr7	117239842	117240528	117237887	65200	Slc16a8
chr7	117566855	117567455	117566504	315131	Kdelr3
chr7	117931790	117932390	117930155	315134	Josd1
chr7	118167516	118168116	118165701	362962	Cbx7
chr7	120225977	120226577	120223146	192246	Phf5a
chr7	120752247	120752847	120752274	171522	Cyp2d4
chr7	120783413	120784013	120783140	24303	Cyp2d3
chr7	122319329	122319929	122322459	689069	Parvg
chr7	137224969	137225569	137223614	24528	Lalba
chr7	137413059	137413659	137412091	300208	Ddx23
chr7	137558322	137559108	137557835	24881	Wnt1
chr7	140811305	140811905	140814662	300253	Eif4b
chr7	141014388	141014988	141014108	685072	Rarg
chr7	21461475	21462075	21461412	299694	Nuak1
chr7	56932210	56932810	56931919	314855	Cpm
chr8	100655577	100656177	100655662	501038	LOC501038
chr8	111573057	111573657	111573458	29283	Rpl29
chr8	111573357	111573957	111573458	29283	Rpl29
chr8	11394350	11394950	11392970	315430	Amotl1
chr8	118350791	118351391	118350134	501083	Pdcd6ip
chr8	120682896	120683496	120680453	81810	Tgfbr2

chr8	124305165	124305765	124303798	301059	Myd88
chr8	124305670	124306270	124303798	301059	Myd88
chr8	21027333	21027933	21029968	300441	Tmem205
chr8	40572492	40573092	40573738	498140	RGD1560888
chr8	52095202	52095802	52094813	353227	Zbtb16
chr8	60673757	60674357	60673400	315696	Snx33
chr8	62361207	62361807	62361346	315716	Cd276
chr8	68371054	68371654	68366801	64302	Rpl4
chr9	10394574	10395174	10396315	316237	Mad2l1bp
chr9	108299861	108300461	108303606	116724	Epb4.1l3
chr9	11053753	11054438	11050948	316241	Nfkbie
chr9	35306502	35307102	35303816	316328	RGD1309220
chr9	35480193	35480793	35479314	316330	Ankrd23
chr9	39492146	39492746	39492186	171106	Il1rl2
chr9	42237153	42237753	42236808	301372	Gpr45
chr9	79165909	79166509	79166330	29366	Serpine2
chr9	8281071	8281671	8282627	301230	RGD1565959
chr9	84373776	84374376	84374287	363269	Sp100
chr9	87051969	87052569	87052168	574523	Ugt1a5
chr9	91718586	91719186	91718819	405023	Olr1345
chr9	91796198	91796798	91797534	405022	Olr1350
chr9	92652623	92653223	92655566	367318	Ano7
chr9	93074370	93074970	93074168	301622	Dtymk
chr9	93187556	93188156	93185687	301626	Pdcd1
chr10	106039395	106039995	106039287	287834	Recql5
chr10	106318772	106319372	106318381	360664	RGD1311078
chr10	106948558	106949158	106948652	360665	Jmjd6
chr10	109317332	109317932	109317686	287873	Chmp6
chr10	109964795	109965395	109961716	25506	P4hb
chr10	12056590	12057190	12059820	58923	Mefv
chr10	13286541	13287141	13288980	287108	Prss27
chr10	13679781	13680381	13682999	29740	Dci
chr10	13991771	13992371	13992928	685059	Sepx1
chr10	14134128	14134728	14134994	287125	Nubp2
chr10	14211586	14212186	14211106	360492	Hn1l
chr10	15492241	15492841	15492467	360502	Itfg3
chr10	27314185	27314785	27313725	29705	Gabra1
chr10	34738266	34738866	34740401	287241	Olr1387
chr10	34748223	34748823	34751126	405066	Olr1388
chr10	36167362	36167962	36168654	287899	Adamts2
chr10	36415685	36416375	36412495	497896	RGD1563273
chr10	36805864	36806464	36808660	353303	Col23a1
chr10	37281198	37281798	37283895	287275	Sec24a
chr10	45237163	45237763	45238122	303175	Hist3h2ba
chr10	46845038	46845638	46845809	54265	Llgl1
chr10	4808722	4809422	4808353	24685	Prm1
chr10	56724861	56725461	56721932	691982	Tmem95
chr10	57193900	57194500	57194388	81639	Alox15
chr10	61512936	61513536	61516479	405994	Olr1515
chr10	63363670	63364270	63363231	497957	RGD1565611
chr10	64611178	64611778	64607332	287545	Sarm1

chr10	67405283	67405883	67407202	94189	Gosr1
chr10	7024085	7024685	7022517	302915	Pmm2
chr10	84451966	84452566	84452883	497984	Znf652
chr10	89383167	89383767	89385133	360629	Nt5c3l
chr10	89562598	89563198	89563723	303536	Dnajc7
chr10	90471685	90472285	90470075	64306	Rpl27
chr10	90493272	90493872	90491463	287721	Vat1
chr10	91363525	91364125	91364902	303567	Tmub2
chr10	92289606	92290206	92290288	498008	Hexim1
chr10	97217513	97218113	97214444	140725	Cacng4
chr11	20422699	20423299	20423191	288280	Ncam2
chr11	33787230	33787830	33785529	245975	Setd4
chr11	43605295	43606009	43604993	304021	Col8a1
chr11	80620258	80620858	80623183	25666	Dgkg
chr11	82425875	82426475	82426105	498109	Polr2h
chr11	84559192	84559792	84561590	24267	Comt
chr12	11198985	11199585	11198145	116693	Cyth3
chr12	12417445	12418141	12420876	304299	Radil
chr12	15825163	15825763	15825016	171097	Adap1
chr12	19692924	19693524	19690356	304374	Fbxo24
chr12	19752379	19752979	19752171	304375	Agfg2
chr12	22722183	22722783	22724526	368190	Dnajc30
chr12	22726579	22727179	22724526	368190	Dnajc30
chr12	2531703	2532303	2529952	304195	Pcp2
chr12	27741478	27742154	27744408	24434	Gusb
chr12	27812784	27813384	27813093	399684	Vkorc1l1
chr12	33288679	33289279	33289889	304470	Sbno1
chr12	33847475	33848166	33847985	689936	Gpr81
chr12	36805324	36805924	36806060	192281	Oas1a
chr12	37336496	37337176	37332257	124451	Lhx5
chr12	42555971	42556661	42552590	288701	Sfrs9
chr12	42579033	42579633	42580541	58945	Dynll1
chr12	42953814	42954414	42951259	288704	RGD1311899
chr12	46024247	46024847	46024977	304567	Pus1
chr13	33464155	33464755	33465845	304743	Ccdc93
chr13	44891786	44892466	44891368	304786	Elk4
chr13	45390825	45391496	45392326	498229	Tmem81
chr13	47092307	47092998	47095239	289032	Chit1
chr13	72785774	72786374	72787821	89868	Sec16b
chr13	76594928	76595528	76594418	304918	Zbtb37
chr13	86914892	86915576	86911743	116591	Fcgr2a
chr13	88249722	88250322	88249075	29132	Atp1a4
chr13	96622608	96623288	96621421	289316	Lefty2
chr13	96987251	96987946	96984348	498301	Wdr26
chr13	99023735	99024335	99025500	289337	Tlr5
chr14	10140785	10141385	10144321	64537	Hpse
chr14	102017601	102018721	102018125	286932	Vps54
chr14	17338187	17338787	17338758	497009	Naaa
chr14	18210047	18210647	18214004	64022	Btc
chr14	3010222	3010822	3007464	305123	Brdt
chr14	37708795	37709395	37708306	305310	Slain2

chr14	42326115	42326715	42329679	360933	LOC360933
chr14	5454401	5455001	5451584	305135	Lrrc8b
chr14	62471157	62471757	62470889	501923	RGD1565192
chr14	83904522	83905122	83905713	305471	Zfp278
chr14	84759189	84759789	84761365	305478	Rnf215
chr14	87000774	87001374	87001869	305502	Tmed4
chr15	22614991	22615591	22617408	289993	Cdkn3
chr15	26854988	26855588	26852308	64523	Tep1
chr15	27348750	27349350	27347196	171114	Ndrg2
chr15	3918438	3919038	3918086	408223	Usp54
chr15	40707189	40707789	40703967	290303	RGD1306437
chr15_random	597707	598307	598165	192264	Ear11
chr16	10030537	10031137	10030513	64347	Sncg
chr16	19039705	19040305	19042062	290641	Rpl18a
chr16	19333956	19334556	19334215	364534	Ssbp4
chr16	19425824	19426424	19425400	64156	Uba52
chr16	19609512	19610112	19609515	290659	Cope
chr16	48728728	48729328	48725513	290749	Irf2
chr16	6359722	6360322	6358767	50693	Itih3
chr16	75113768	75114368	75116871	613224	Defa8
chr17	12196904	12197504	12196629	117282	Hnrnpk
chr17	15351908	15352508	15353097	361208	Rab24
chr17	16084231	16084831	16084777	290999	Higd2a
chr17	21188357	21188957	21185525	306808	Ippk
chr17	23817293	23817893	23819083	308173	Kif13a
chr17	32839813	32840413	32840752	291060	Snrnp48
chr17	48513464	48514064	48513673	498753	RGD1564767
chr17	5549357	5549957	5549458	306687	Hiatl1
chr17	83395155	83395755	83396733	361272	Dhtkd1
chr17	90572122	90572722	90572390	361282	Plxdc2
chr18	29154450	29155129	29153944	25433	Hbegf
chr18	29729968	29730660	29728602	394223	Pcdha11
chr18	30150433	30151033	30150686	291653	Pcdhb6
chr18	30670860	30671460	30669477	364845	Pcdhgb8
chr18	71887964	71888564	71889052	291433	Dym
chr18	75051310	75051910	75048481	54301	Slc14a1
chr18	77135010	77135610	77134655	100192205	LOC100192205
chr18	81233561	81234161	81232949	291394	Cndp2
chr18	83307981	83308686	83306944	291388	Cbln2
chr19	11289146	11289746	11286402	117038	Mt3
chr19	20438304	20438904	20439008	94188	Zfp423
chr19	21714297	21714897	21717140	140941	Siah1a
chr19	40870230	40870830	40873201	64442	St3gal2
chr19	45861256	45861856	45861454	54267	Maf
chr19	50464867	50465467	50465553	307907	RGD1304884
chr19	54479100	54479700	54479960	292090	Galnt2
chr19	55935100	55935700	55939044	361443	RGD1306192
chr20	10973196	10973796	10973422	309680	Dnmt3l
chr20	12086996	12087596	12089172	294336	Pcbp3
chr20	1658131	1658731	1658454	414785	RT1-M6-1
chr20	3539070	3539670	3539612	414783	RT1-CE4

chr20	3554455	3555135	3555613	414793	RT1-CE3
chr20	3555556	3556156	3555613	414793	RT1-CE3
chr20	3797372	3797972	3797594	361796	Bat5
chrX	134397140	134397818	134396762	58812	Apln
chrX	25481044	25481644	25480915	363448	Dynlt3
chrX	26711844	26712444	26710222	116695	Kcnd1
chrX	80627745	80628345	80631374	317268	Arx
chrX	94138301	94138901	94138055	303393	Cox7b
chrX	94353841	94354441	94351923	171152	Taf9b
