# Supplementary material for: Pesticide Methoxychlor Promotes the Epigenetic Transgenerational Inheritance of Adult-Onset Disease through the Female Germline
Source: PLoS One. 2014 Jul 24;9(7):e102091. doi: 10.1371/journal.pone.0102091 (PMC4109920; doi:10.1371/journal.pone.0102091)
Supplement: Table S8 — Characteristics of the average epimutation clusters and associated genes. Clusters with the same gene listed more than once indicates multiple epimutations associated with that gene. (PDF) [file pone.0102091.s011.pdf]

Supplemental Table S8

Characteristics of the Average Epimutation Clusters and Associated Genes

Chromosome Cluster	cSTART	cSTOP	Epimutation Associated Genes in Cluster
chr1	77950000	81900000	Qpctl, RGD1565787, Lypd3, Cyp2b1, Cyp2b12
chr1	207900000	209950000	Cst6, Mrpl49, Cox8a
chr1	209650000	211800000	Cox8a, Tut1, Tut1
chr3	71950000	75450000	Olr636, Olr681, Olr705, Mtch2
chr3	168100000	171250000	Rps21, Ntsr1, Nkain4
chr5	158750000	162550000	Ddi2, Agmat, Dnajc16
chr7	8350000	10900000	Zbtb7a, Thop1, Timm13
chr7	10150000	12900000	Timm13, Apc2, Apc2, Apc2
chr7	115950000	119550000	Slc16a8, Kdelr3, Josd1, Cbx7
chr7	118800000	122750000	Phf5a, Cyp2d4, Cyp2d3, Parvg
chr7	135600000	139150000	Lalba, Ddx23, Wnt1
chr9	90700000	94600000	Olr1345, Olr1350, Ano7, Dtymk, Pdcd1
chr10	11700000	14200000	Mefv, Prss27, Dci, Sepx1, Nubp2
chr10	13300000	16100000	Dci, Sepx1, Nubp2, Hn1l, Itfg3
chr10	34200000	38400000	Olr1387, Olr1388, Adamts2, RGD1563273, Col23a1, Sec24a
chr10	88500000	92450000	Nt5c3l, Dnajc7, Rpl27, Vat1, Tmub2, Hexim1
chr10	104950000	107950000	Recql5, RGD1311078, Jmjd6
chr12	41000000	44500000	Sfrs9, Dynll1, RGD1311899
chr16	17450000	21250000	Rpl18a, Ssbp4, Uba52, Cope
chr18	28200000	31650000	Hbegf, Pcdha11, Pcdhb6, Pcdhgb8
chr20	1600000	5500000	RT1-M6-1, RT1-CE4, RT1-CE3, RT1-CE3, Bat5

Clusters with the same gene listed more than once indicates multiple epimutations associated with that gene.
